# HBV immune tolerance of HBs-transgenic mice observed through parabiosis with WT mice

**DOI:** 10.3389/fimmu.2022.993246

**Published:** 2022-09-20

**Authors:** Wendi Zhang, Haoyu Sun, Rui Sun, Zhexiong Lian, Haiming Wei, Zhigang Tian, Yongyan Chen

**Affiliations:** ^1^ Hefei National Laboratory for Physical Sciences at Microscale, the CAS Key Laboratory of Innate Immunity and Chronic Disease, School of Basic Medical Sciences, Division of Life Sciences and Medicine, University of Science and Technology of China, Hefei, China; ^2^ Institute of Immunology, University of Science and Technology of China, Hefei, China

**Keywords:** hepatitis B virus, CD8^+^ T cell, CD4^+^ T cell, regulatory T Cell, parabiotic mice model

## Abstract

It was extensively recognized that central tolerance to HBV exists in HBs-transgenic (Tg) mice, however, the immune response to HBV vaccine may be inspired in adult HBs-Tg mice after boosting with potent adjuvants, leaving a mystery to explore its immune tolerance. Here, WT-HBs-Tg parabiotic mice model was generated by conjoining WT (donor) and HBs-Tg (host) mouse *via* parabiotic surgery, in order to see how immunocompetent WT mice naturally respond to HBV, and how tolerant HBs-Tg mice influence the anti-HBV immunity from WT mice. It was found that WT CD8^+^ T cells markedly accumulated into the liver of HBs-Tg parabionts, and importantly, almost all HBsAg-specific CD8^+^ T cells derived from WT but not HBs-Tg mice, making a clear separation of a normal immune response from WT donor and a tolerant response by recipient host. Further, in the absence of host but not donor spleen, HBsAg-specific CD8^+^ T cells disappeared, indicating that host spleen was the indispensable site for donor HBsAg-specific CD8^+^ T cell priming though its mechanisms need further study. We found that donor CD4^+^ T helper cells were necessary for donor HBsAg-specific CD8^+^ T cell response by CD4-deficiency in WT or in HBs-Tg mice, indicating that an immune response was elicited between CD4^+^ T helper cells and CD8^+^ cytotoxic T cells of donor in the host but not donor spleen. It was noted that compared to donor CD4^+^ T cells, host CD4^+^ T cells were characterized with more tolerant features by harboring more CD25^+^Foxp3^+^ Tregs with higher expression of PD-1 and TIGIT in the spleen of HBs-Tg parabionts, which exhibited suppressive function on CD8^+^ T cells directly. Moreover, the Th1/Treg ratio was enhanced after parabiosis, suggesting that donor T helper cells may overcome the negative regulation of host Tregs in host spleen. In conclusion, both incompetent anti-HBV CD8^+^ T cells and insufficient help from CD4^+^ T cells are the major mechanisms underlying immune tolerance in HBs-Tg mice which helps explain HBV persistence.

## Introduction

Chronic hepatitis B virus (HBV) infection has been a health concern worldwide with a risk of developing hepatitis, liver fibrosis, liver cirrhosis, and hepatocellular carcinoma (HCC) (1). According to World Health Organization (WHO) reports, approximately 290 million people have been chronically infected with HBV, among which about 1.5 million new cases each year (2). More than 90% of neonates and 30% of children (1-5 years) usually develop a persistent infection when exposed to HBV due to their host immune tolerance, whereas approximately 95% of infected adults spontaneously clear the virus owing to their effective adaptive immune responses (1, 3, 4). Thus, the host immune response to HBV is a key determinant of the outcome of HBV infection. Reversal of host immune tolerance is widely considered to be a good therapeutic strategy to achieve the functional cure of chronic HBV infection (5, 6). However, the precise mechanisms of HBV immune tolerance are not well understood, which is urgent to be elucidated.

HBV-specific CD8^+^ T cells are pivotal in antiviral activity either by indirectly secreting cytokines, such as IFN-γ and TNF-α, or directly killing HBV infected hepatocytes, but their response is weak in chronic hepatitis B (CHB) patients exhibiting immune tolerance (7). Recently, evidence showed that mMDSCs cross-presenting HBsAg migrated to the thymus and selectively deleted HBsAg-specific CD8^+^ thymocytes in infants and young patients, resulting in CD8^+^ T cell tolerance and HBV persistence (8). Moreover, HBV-specific CD8^+^ T cells upregulated TRAIL-R2 and became susceptible to NK cell-mediated killing, which contributed to the failure of antiviral immunity in CHB patients (9). However, numerous HBV core-specific and HBV polymerase-specific CD8^+^ T cells with polyfunctionality and long-term memory were detected in chronic HBV-infected patients (10–12). Conversely, HBV-specific CD8^+^ T cells were demonstrated to be exhausted CD8^+^ T cells, exhibiting several kinds of inhibitory receptors such as PD-1, CTLA-4, Tim-3, and TIGIT in chronic HBV infection (7, 13–15). Additionally, HBV antigen-presentation by intrahepatic APCs with weakened costimulatory signals also contributed to peripheral tolerance of HBV-specific CD8^+^ T cells during chronic HBV infection (15). Potent adjuvants such as Poly I: C, GM-CSF, CpG, and TLR7/8 agonist, can effectively induce DC maturation and then inspire the HBV-specific CD8^+^ T cell response, leading to overcoming immune tolerance in chronic HBV infection (16–19). These data highlight that CD8^+^ T cell immune tolerance can be induced by multiple mechanisms including clone deletion, functional suppression, or clone anergy. In order to induce effective HBV-specific CD8^+^ T cells, the mystery veil of immune tolerance needs to be revealed thoroughly.

CD4^+^ T cells are pivotal for the priming of CD8^+^ T cells and maintenance of CD8^+^ T cell effector function (20). In chimpanzee and mouse models of acute HBV infection, the depletion of CD4^+^ T cells before or during the initial phase of infection prevented CD8^+^ T cell priming and impaired the CD8^+^ T cell response, therefore leading to persistent HBV infection (21, 22). However, depletion of CD4^+^ T cells after HBV viral spread massively did not affect the quality of the CD8^+^ T cell response to HBV (23). Loss of the immunodominant CD8^+^ T cell responses and insufficient CD4^+^ T cell help to CD8^+^ T cells contributed to HBV persistence in patients (24). Restoring systemic HBV-specific CD4^+^ T cell responses could elicit vigorous hepatic HBV-specific CD8^+^ T cell responses in HBV-carrier mice (25). Thus, the absence or paucity of CD4^+^ T cell help might be one of the important mechanisms of CD8^+^ T cell tolerance or dysfunction.

A higher frequency of CD4^+^CD25^+^Foxp3^+^ regulatory T cells (Tregs) was observed in patients with chronic HBV infection compared with individuals with resolved HBV infection and healthy controls, showing immunosuppression on HBV-specific T helper cells (26). IL-10-secreting HBcAg-specific Tregs might contribute to the suppression of the HBcAg-specific Th1 cell response (27). The suppression of Tregs could be overcome by enhanced Th1 activities, for example vaccination with adjuvants or IL-12 against HBV (25, 28). In addition, Tregs could directly suppress the function of effector CD8^+^ T cells including their proliferation and production of IFN-γ in CHB patients (29). By using HBV animal models, Tregs were demonstrated to limit IFN-γ and TNF-α production and cytotoxicity of HBV-specific CD8^+^ T cells, rather than the development of HBV-specific CD8^+^ T cells or memory T cells (30, 31). Conversely, Tregs suppressed the priming of HBV-specific CD8^+^ T cells during DNA immunization (32). It remains controversial about Treg regulations on HBV-specific CD8^+^ T cells. Moreover, the balance between HBV-specific Th1 cells and Tregs during HBV infection and their regulations on HBV-specific CD8^+^ T cell response deserve further investigation.

Owing to HBV immune tolerance of HBV transgenic mice, exploration of the precise mechanisms of HBV immune responses and regulations have been limited. We previously reported a new HBV mouse model in which HBsAg^+^ hepatocytes from HBs-Tg mice were transferred into immunocompetent Fah^-/-^ mice (33). HBsAg-specific CD8^+^ T cells were generated, but the damage and regeneration of hepatocytes in recipient mice may cause liver inflammation and cancer which are unrelated to HBV (34). Thus, a model to see how the immune system naturally responds to HBV and how to influence HBV tolerance is necessary. In this study, we developed a new mouse model by conjoining WT (donor) and HBs-Tg (host) mouse *via* parabiotic surgery, in which both parabionts shared common circulating HBsAg and cell migration by blood transportation. In this model, HBsAg persistently exists and there is no other cause of death leading to liver inflammation except for the HBsAg-specific immune response. The two systems: one is donor HBV-naïve immune system; the other is host HBV-tolerant immune system, can preferably help us to see how immunocompetent WT mice naturally respond to HBV, and how tolerant HBs-Tg mice influence the anti-HBV immunity from WT mice. It was found that HBsAg-specific CD8^+^ T cells were derived from donor WT mice but not host HBs-Tg mice, and the spleen of HBs-Tg mice was a normal priming site for CD8^+^ T cells in the presence of donor CD4^+^ T helper cells, which overcame the negative regulation of host Tregs. Both incompetent anti-HBV CD8^+^ T cells and insufficient help from CD4^+^ T cells are the major mechanisms underlying immune tolerance in HBs-Tg mice, which provides a theoretical basis for promoting an effective CD8^+^ T cell response in chronic HBV infection.

## Materials and methods

### Mice

8 to 12-week-old male HBV transgenic mice C57BL/6J-TgN (Alb-1 HBV) 44Bri (HBs-Tg mice), were purchased from the Department of Laboratory Animal Science of Peking University (Beijing, China). CD45.1^+^ mice were purchased from The Jackson Laboratory (Bar Harbor, ME) and used to obtain CD45.1^+^HBs-Tg mice. CD4^-/-^ mice were a gift from Dr. Zhexiong Lian (University of Science and Technology of China) and used to obtain CD4^-/-^HBs-Tg mice. All mice were housed under specific-pathogen-free (SPF) conditions and used in accordance with the guidelines outlined in the Guide for Experimental Animals at the University of Science and Technology of China.

### Parabiotic surgery

Parabiosis surgeries were performed as previously described (35, 36). In brief, well-matched (sex, age and weight) mouse partners were anesthetized with 1% pentobarbital sodium (50 mg/kg) by intraperitoneal injection, and then shaved and sterilized. The lateral skin of each mouse was incised from the olecranon to the knee joint. Then the olecranons and knee joints were attached and tied with non-absorbable 3-0 silk suture, and the dorsal and ventral skins were stitched with a continuous suture starting ventrally from the elbow towards the knee. The parabiotic mice were placed on heating pad until recovery. Prophylactically, sulfamethoxazole/trimethoprim oral suspension in their water bottle (2 mg sulfa/mL + 0.4 mg trim/mL) was administered to parabiotic mice for 10 days to prevent bacterial infections. Then, parabiotic pairs were analyzed 4 weeks after surgery.

### Splenectomy surgery

Mice were anesthetized with 1% pentobarbital sodium (50 mg/kg) by intraperitoneal injection. The mouse abdomen was shaved and sterilized, and then was made with a 1 cm laparotomy incision. The spleen was exposed, and then splenic vessels were ligated with 3-0 silk suture. Next, the spleen was removed, and the abdomen was stitched with the absorbable 4-0 silk suture.

### Parabiotic model

HBs-Tg mice (CD45.2^+^) were conjoined with WT mice (CD45.1^+^) by parabiosis surgery to generate WT-HBs-Tg parabiotic mice. In this model, HBs-Tg mice were considered as hosts, and WT mice were considered as donors. WT mice (CD45.1^+^) were performed splenectomy (Sx), and then surgically conjoined with HBs-Tg (CD45.2^+^) mice to generate WT-Sx-HBs-Tg parabiotic mice. HBs-Tg mice (CD45.2^+^) were performed splenectomy, and then conjoined surgically with WT mice (CD45.1^+^) to generate WT-HBs-Tg-Sx parabiotic mice. HBs-Tg mice (CD45.1^+^) were surgically conjoined with CD4^-/-^ mice (CD45.2^+^) to generate CD4^-/-^-HBs-Tg parabiotic mice. CD4^-/-^HBs-Tg mice (CD45.2^+^) were surgically conjoined with WT mice (CD45.1^+^) to generate WT-CD4^-/-^HBs-Tg parabiotic mice.

### Isolation of mononuclear cells

In brief, the liver samples were harvested and passed through a 200-gauge mesh and collected with centrifugation. Then the pellet was suspended with 40% Percoll (GE Healthcare, Chicago, IL, USA) and overlaid onto 70% Percoll solutions with centrifugation at 750 g for 30 min at room temperature. Then, liver MNCs were collected from the interphase of the two Percoll gradients after centrifugation. PBMCs were isolated from peripheral blood by red blood cell (RBC) lysis solution (Solarbio, Beijing, China) for 10 min at 4°C and then collected with centrifugation at 450 g for 10 min at 4°C. The spleen samples were harvested and passed through a 200-gauge mesh. Splenocytes were treated with RBC lysis solution and collected with centrifugation at 250 g for 10 min.

### Antibody staining and flow cytometry analysis

MNCs were incubated with rat serum for 30 min at 4°C to block Fc receptors and then stained with the indicated fluorescently labeled antibodies at 4°C for 30 min in darkness. For the intracellular assay, isolated MNCs were stimulated with 30 ng/mL phorbol 12-myristate 13-acetate (PMA) (Sigma–Aldrich, Saint Louis, MO, USA) and 1 μg/mL ionomycin (Sigma–Aldrich) and treated with 2.5 μg/mL monensin (Sigma–Aldrich) for 4 h. Next stain for surface antigens at 4°C for 30 min in darkness. The cells were fixed and permeabilized using 100 μL of Fixation buffer (Thermo Fisher Scientific, Carlsbad, CA, USA) for 1 h at 4°C and washed with 1×Permeabilization buffer (Thermo Fisher Scientific). Finally, the cells were stained with intracellular antibodies for 1 h at 4°C in darkness. For staining of Foxp3 and CTLA-4, cells were fixed and permeabilized, and then stained with the indicated antibodies. An LSR Fortessa flow cytometer (BD Biosciences, San Diego, CA, USA) was used and the data were analyzed using FlowJo (BD Biosciences).

The mAbs included BUV395-CD3e (145-2C11), BUV563-CD4 (GK1.5), BUV737-CD8α (53-6.7), BV510-CD19 (1D3), BV510-CD4 (RM4-5), PerCP-Cy5.5-CD44 (IM7), PE-CD8α (53-6.7), PE-CD69 (H1.2F3), PE-CTLA-4 (VC10-4F10-11), FITC-CD25 (7D4), FITC-CD62L (MEL-14), FITC-TNFα (MP6-XT22), BV786-CD45.2 (104), and BV786-IFN-γ (XMG1.2) (BD Biosciences); PE-Granzyme B (QA16A02), PE-CD137 (17B5), FITC-CD8α (5H10-1), APC-CD4 (RM4-4), APC-KLRG1 (2F1/KLRG1), BV421-Tim-3 (RMT3-23), BV421-CD28 (37.51), BV421-CD127 (A7R34), BV421-CD45.1 (A20), BV421-IL-2 (JES6-5H4), BV605-NK1.1 (PK136), BV785-CD69 (H1.2F3), BV785-PD-1 (29F.1A12), APC-Cy7-CD45.1 (A20), APC-Cy7-CD45.2 (104), and PE-Cy7-CD45.1 (A20) (BioLegend, San Diego, CA, USA); APC-TCRγδ (eBioGL3); eFluor 660-Ki67 (SolA15), eFluor 660-TIGIT (GIGD7), and PerCP-Cy5.5-Foxp3 (FJK-16s) (eBioscience, San Diego, CA, USA).

### Detection of HBsAg-specific CD8^+^ T cells

Freshly isolated liver MNCs were resuspended in a total volume of 50 μL phosphate buffer saline (PBS) (pH 7.4) containing 5% fetal calf serum (Gibco, Carlsbad, UT, USA), and stained with 10 μL HBsAg-dextramer (FITC-H-2k^b^-HBsAg_VWLSVIWM_) (JD3577-FITC, Immudex, Copenhagen, Denmark) for 10 min at room temperature in darkness. Then cells were stained with other surface antibodies for 20 min at 4°C in darkness. Cells were washed with PBS and stained with 7-AAD (BD Biosciences) for 5 min at room temperature. Samples were processed on LSR Fortessa flow cytometer (BD Biosciences) and data were analyzed with FlowJo (BD Biosciences).

### Serum transaminase activity assays

Serum alanine aminotransferase (ALT) and aspartate aminotransferase (AST) activities were measured by standard photometric methods using a kit (DIRUI, Changchun, China) and detected by a Bio-Chemical Analyzer (Rayto, Shenzhen, China). Due to the twice-increased volume of circulating blood, the measured values of ALT and AST were multiplied by 2 to be considered as actual values in HBs-Tg parabionts.

### Histological analysis

Liver samples were fixed in 4% paraformaldehyde and embedded in paraffin. Liver sections (4 μm) were dewaxed, rehydrated and stained with hematoxylin and eosin (H&E) using routine methods. The number of inflammatory foci was calculated per field (at a magnification of 20×) in 5 random fields in each sample. For immunohistochemistry (IHC) analysis, liver sections were dewaxed, rehydrated, and heat retrieval by citrate acid solution (pH 6.0) for 15 min. Then primary antibody against CD8α (1:200, Cat#98941, Cell Signaling Technology, Danvers, MA, USA) was incubated overnight. Signal detections were performed using the DAB Peroxidase Substrate Kit (SK-4100, Vector Laboratories). The sections were photographed using a Pannoramic MIDI or SCAN (3D HISTECH, Budapest, Hungary) with CaseViewer software. The number of CD8 positive cells was calculated per field (at a magnification of 20×) in 5 random fields in each sample.

### *In vitro* Treg suppression assay

CD4^+^CD25^+^ Tregs were isolated from the spleens of HBs-Tg mice by using a CD4^+^CD25^+^ Regulatory T Cell Isolation Kit (Cat#130-091-041, Miltenyi Biotec, Bergisch Gladbach, Germany). CD8^+^ T cells were isolated from the spleens of WT B6 mice (CD45.1^+^) by using MACS microbeads (Cat#130-048-801, Miltenyi Biotec). Purified CD8^+^ T cells were labeled with 5 μM CellTrace Violet (Invitrogen, Eugene, Oregon, USA). The CTV-labeled CD8^+^ T cells were plated on 96-well plate (1×10^5^/well) or 24-well plate (1×10^6^/well), and co-cultured with CD4^+^CD25^+^ Tregs in the presence of anti-CD3 mAb (2 μg/mL; BD Biosciences), anti-CD28 mAb (1 μg/mL; BD Biosciences), and IL-2 (100 IU/mL; Jiangsu Kingsley Pharmaceutical, China) for 3 days. Transwell membranes (0.4-μm pore size) were inserted into 24-well plate to separate cell-cell contact between CD8^+^ T cells and Tregs. For the blockade, anti-TIGIT mAb (clone: 13G6) was purified in-house from hybridoma cell supernatants and used (10 μg/mL). Rat IgG isotype control was purified in-house from rat serum (D9018; Yaanda Biology, Beijing, China) and used as the control (10 μg/mL). The proliferation of CD8^+^ T cells was analyzed by using flow cytometry. The inhibition activity was calculated as follows (37): % inhibition = (control proliferation of CD8^+^ T cells - sample proliferation of CD8^+^ T cells)/control proliferation of CD8^+^ T cells ×100.

### Statistical analysis

Statistical significance was determined in GraphPad PrisimV6.02 using appropriate tests as indicated. An unpaired two-tailed t-test or one-way analysis of variance (ANOVA) with Sidak’s multiple comparison test was used to compare the experimental groups. A Fisher’s exact test was used to compare the positive rate of MHC dextramer staining in different parabionts. The Mann-Whitney test was used to compare the frequency and number of HBsAg dextramer^+^ CD8^+^ T cells between the two groups. Two-way ANOVA with Sidak’s multiple comparison test was used to compare the proportion of CD45.1^+^ (donor) or CD45.2^+^ (host) in HBsAg dextramer^+^ CD8^+^ T cells. *P* < 0.05 indicated a significant difference.

## Results

### WT CD8^+^ T cells markedly accumulate in the liver of HBs-Tg parabionts

**Figure 1:**
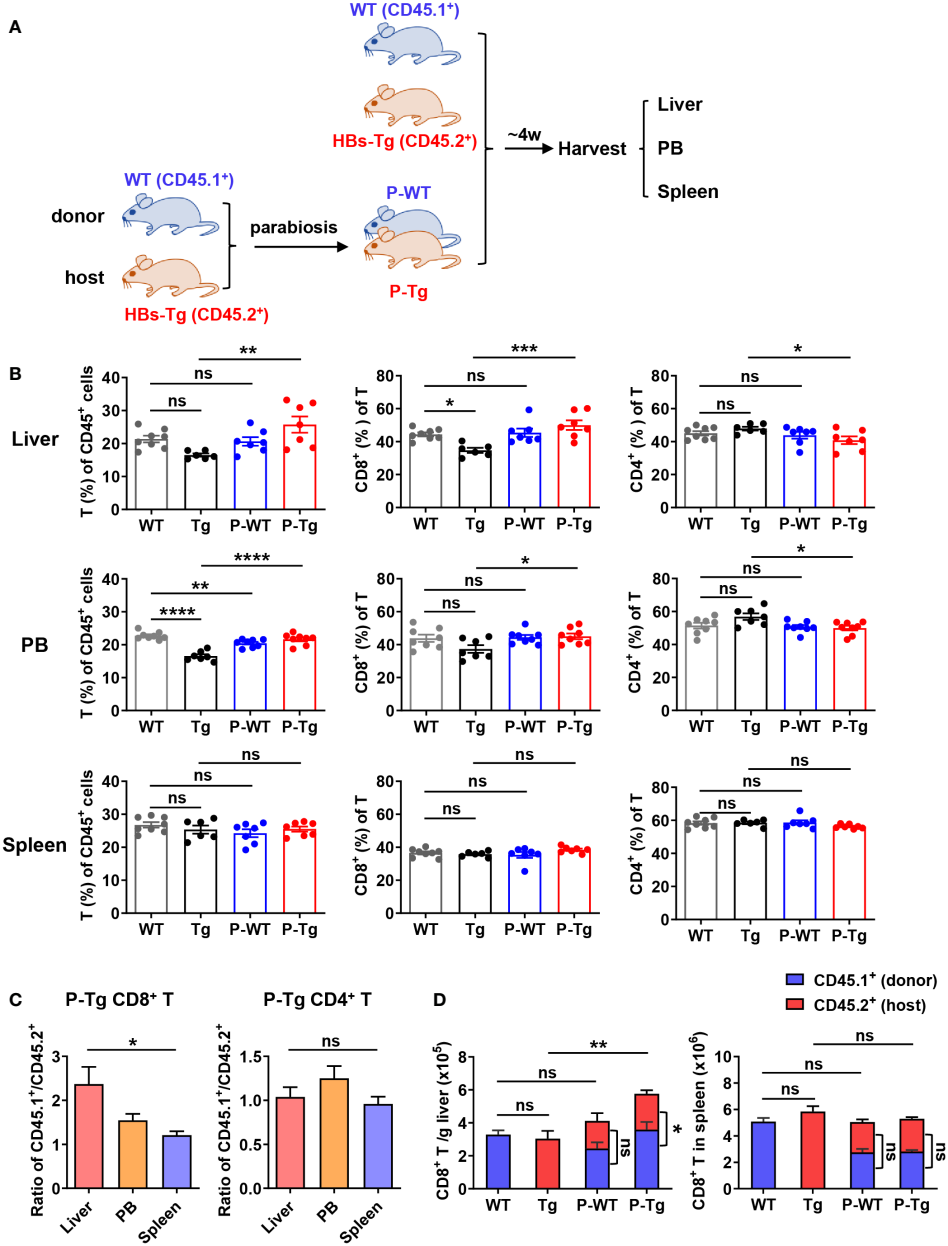
Increased CD8^+^ T cells were predominantly derived from donor in the liver of HBs-Tg parabionts. HBs-Tg mice (CD45.2^+^) were conjoined with WT mice (CD45.1^+^) by parabiosis surgery to generate WT-HBs-Tg parabiotic mice. After 4 weeks of surgery, parabiotic mice were separated and then liver, peripheral blood (PB) and spleen samples were harvested to examine the chimerisms. Mononuclear cells (MNCs) were isolated for flow cytometry analysis. **(A)** Protocol for the model of WT-HBs-Tg parabiotic mice (schematic). **(B)** The frequencies of T cells (CD45^+^ CD3^+^NK1.1^-^) of CD45^+^ cells, and the percentages of CD8^+^ T and CD4^+^ T of total T cells were shown in the liver, peripheral blood and spleen at 4 weeks after parabiosis. **(C)** The ratio of CD45.1^+^ (donor)/CD45.2^+^ (host) in CD8^+^ T and CD4^+^ T cells from liver, peripheral blood and spleen of the HBs-Tg parabionts. **(D)** The number of CD45.1^+^CD8^+^ T cells (donor) and CD45.2^+^CD8^+^ T cells (host) in the liver and spleen. Data are shown as mean ± SEM. One-way ANOVA with Sidak’s multiple comparison test was used to compare the experimental groups **(B, C)**. An unpaired two-tailed t-test was used to compare the number of CD45.1^+^ cells and CD45.2^+^ cells for CD8^+^ T cells in P-WT and P-Tg, respectively **(D)**. One-way ANOVA with Sidak’s multiple comparison test was used to compare the number of total CD8^+^ T cells between WT and P-WT, Tg and P-Tg **(D)**. *P < 0.05; **P < 0.01; ***P < 0.001; ****P < 0.0001; ns, not significant. WT, WT control; Tg, HBs-Tg control; P-WT, WT parabiont; P-Tg, HBs-Tg parabiont; PB, peripheral blood.

It was noted that compared with WT mice, the frequency of CD8^+^ T cells from HBs-Tg mice was slightly decreased in the liver but not the spleen, while there was no change in the frequency of CD4^+^ T cells in either the liver or the spleen of HBs-Tg mice (**Figure S1**). Moreover, higher expression levels of inhibitory molecule TIGIT and terminal effector-like marker KLRG1, but decreased expression levels of co-stimulatory molecule CD137 and memory-like phenotype maker CD127 on intrahepatic CD8^+^ T and CD4^+^ T cells were observed in HBs-Tg mice, indicating intrahepatic CD8^+^ T and CD4^+^ T cells were dysfunctional in HBs-Tg mice (**Figure S2**). To investigate the immune response to HBV more precisely *in vivo*, a model of parabiotic mice was used. As shown in **Figure 1A**, two kinds of syngeneic mice: donor WT mouse (CD45.1^+^) and host HBs-Tg mouse (CD45.2^+^), were conjoined by parabiotic surgery to generate WT-HBs-Tg parabiotic mice. In this model, both parabionts shared common circulating HBsAg and cell migration by blood transportation. The HBs-Tg parabiont had two systems: one from the donor naïve immune system to HBV; the other from its own HBV-tolerant immune system.

After 4 weeks surgery, parabiotic mice were surgically separated for the analysis of immune cell populations in the liver, peripheral blood and spleen. There were no significant differences in the frequencies of innate immune cells including NK, NKT, and γδT cells in the liver and peripheral blood of HBs-Tg parabionts compared with the control, and only a slight increase in the frequency of NKT cells was observed in the spleen of HBs-Tg parabionts (**Figure S3**). The frequency of T cells (CD45^+^CD3^+^NK1.1^-^) in HBs-Tg parabionts (P-Tg) was significantly increased in the liver and peripheral blood but not the spleen (**Figure 1B**). Accordingly, the proportion of CD8^+^ T cells was markedly increased, however, the proportion of CD4^+^ T cells was decreased (**Figure 1B**). Furthermore, an uneven distribution of CD8^+^ T cells but not CD4^+^ T cells to parabiotic partners was observed, as shown by the higher ratio of CD45.1^+^/CD45.2^+^ CD8^+^ T cells in the liver than in the spleen (**Figure 1C**), indicating that donor CD8^+^ T cells might predominantly migrate into the liver of HBs-Tg parabionts. Total amounts of CD8^+^ T cells were markedly increased in the liver of HBs-Tg parabionts, among which donor CD45.1^+^CD8^+^ T cells occupied much more in the liver of HBs-Tg parabionts (**Figure 1D**), further demonstrating that the accumulated CD8^+^ T cells in the liver of HBs-Tg parabionts were mostly derived from donor WT mice. These significant changes were not observed in the spleen of HBs-Tg mice after parabiosis (**Figures 1B, D**). Notably, a higher proportion of CD44^+^CD62L^-^ effector memory CD8^+^ T cells and increased expression of CD69, Ki67 and Granzyme B in these CD8^+^ T cells were observed in the liver of HBs-Tg parabionts, indicating their stronger proliferation and cytotoxicity (**Figure S4A**). It was also noted that CD4^+^ T cells were markedly activated in the spleen of both WT and HBs-Tg parabionts with a CD44^+^CD62L^-^ effector memory phenotype (**Figure S4B**).

**Figure 2:**
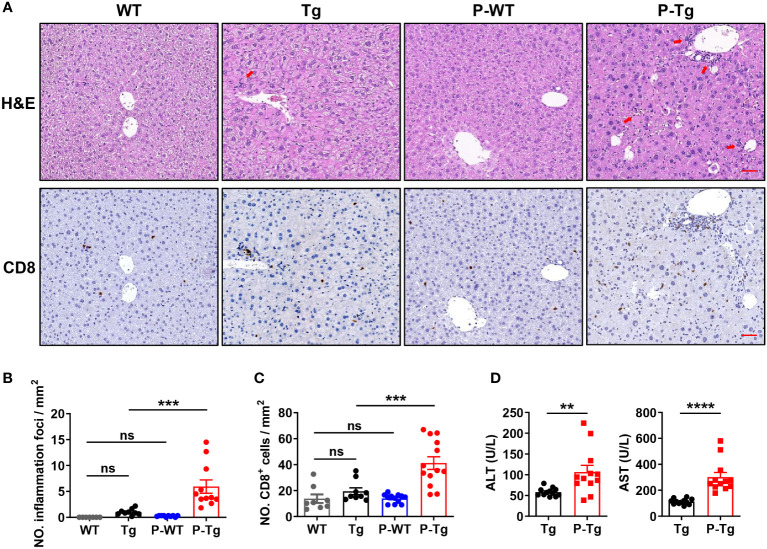
Inflammation foci with accumulated CD8^+^ T cells were observed in the liver of HBs-Tg parabionts. WT-HBs-Tg parabiotic mice were generated by parabiosis surgery as shown in **Figure 1A**. Four weeks after parabiosis, liver samples were harvested. **(A)** Representative H&E staining of liver tissue. Inflammation foci are shown by red arrows. CD8^+^ cells in the liver tissue were detected by immunochemistry analysis. Dark brown dots represent positive staining. Scale bar = 50 μm. **(B)** The number of inflammation foci was calculated per field (20×). Average of five random fields in each sample, 7-10 samples/group. **(C)** The numbers of CD8^+^ cells were calculated per field (20×). Average of five random fields in each sample, 8-12 samples/group. **(D)** Serum levels of ALT and AST of the HBs-Tg parabionts. Data are shown as mean ± SEM. One-way ANOVA with Sidak’s multiple comparison test was used to compare the experimental groups **(B, C)**. An unpaired two-tailed t-test was used to compare the experimental groups **(D)**. **P < 0.01; ***P < 0.001; ****P < 0.0001; ns, not significant. WT, WT control; Tg, HBs-Tg control; P-WT, WT parabiont; P-Tg, HBs-Tg parabiont.

Further, more inflammatory foci by H&E staining were observed in the liver of HBs-Tg but not WT parabionts (**Figures 2A, B**). Notably, more CD8^+^ cells were accumulated in these inflammatory foci by IHC staining (**Figure 2A**) and the increased number was further confirmed in the liver of HBs-Tg parabionts (**Figure 2C**). Higher levels of serum ALT and AST in HBs-Tg parabionts were demonstrated, indicating the chronic inflammatory injury in the HBs-Tg parabionts (**Figure 2D**). Thus, CD8^+^ T cells were markedly accumulated and activated in the liver of HBs-Tg parabionts.

### Effective anti-HBV CD8^+^ T cell responses can be elicited in liver of HBs-Tg parabionts

**Figure 3:**
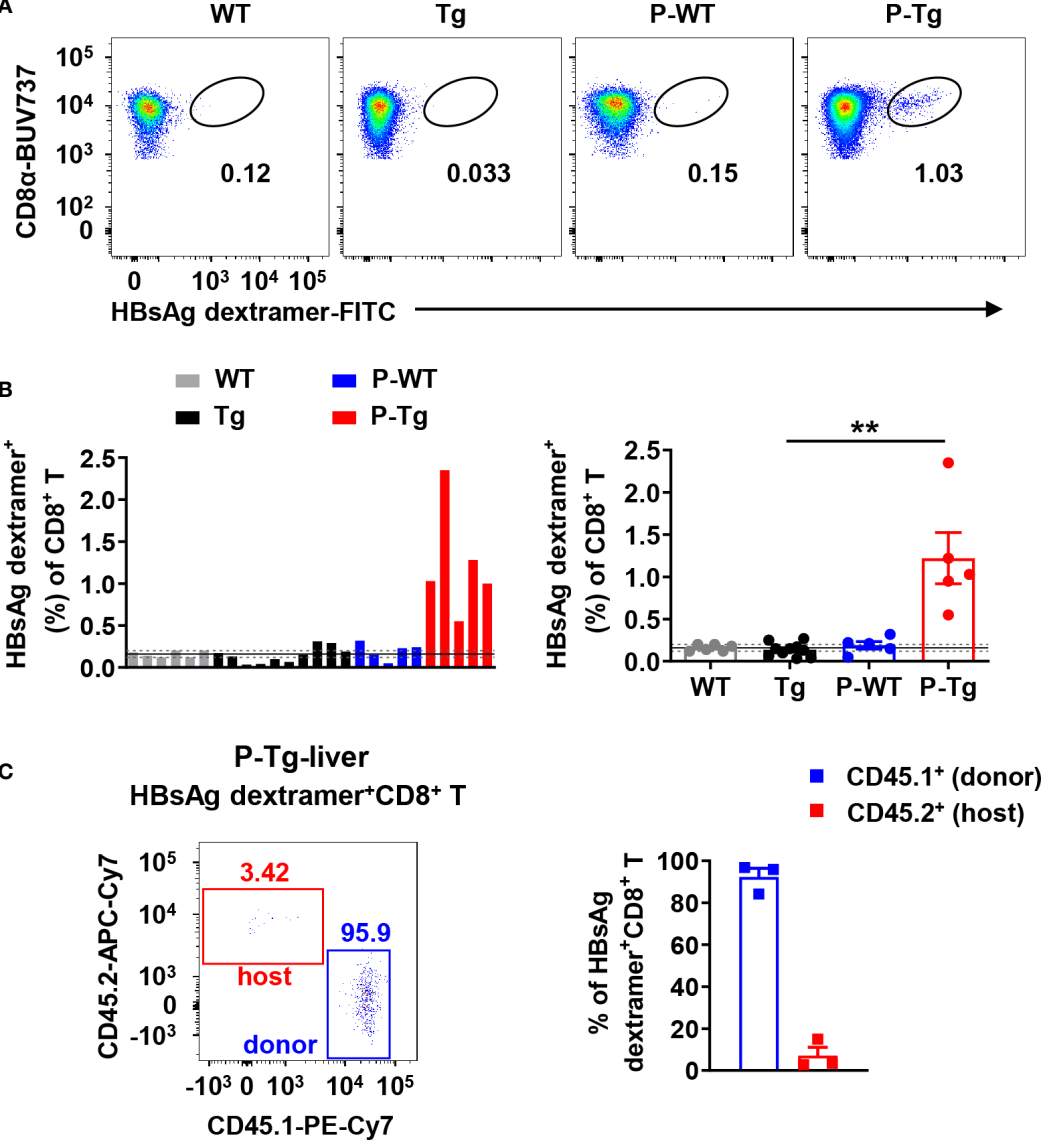
Intrahepatic HBsAg-specific CD8^+^ T cells in HBs-Tg parabionts were derived from donor WT mice but not host HBs-Tg mice. HBs-Tg mice (CD45.2^+^) were conjoined with WT mice (CD45.1^+^) to generate WT-HBs-Tg parabiotic mice. Four weeks after parabiosis surgery, parabiotic mice were separated and liver samples were harvested from each group. Intrahepatic MNCs were isolated for flow cytometry analysis. **(A)** Representative plots showing the frequency of H-2^kb^-HBsAg_VWLSVIWM_ dextramer^+^ CD8^+^ T cells in the liver of each group. CD8^+^ T cells (CD45^+^ CD3^+^NK1.1^-^CD8^+^) were gated to analyze the HBsAg-specific CD8^+^ T cells by using MHC dextramer staining. **(B)** The frequency of H-2^kb^-HBsAg_VWLSVIWM_ dextramer^+^ CD8^+^ T cells in each sample was shown. According to the data of the control WT group (negative control), the mean value (black line, 0.16%) and 95% confidence interval (CI) (gray dotted line, 0.16% ± 0.04%, n=6) were obtained, indicating that the data of the frequency of HBsAg dextramer^+^ CD8^+^ T cells (> 0.20%) were considered statistically positive. Statistical analysis of the frequency of HBsAg dextramer^+^ CD8^+^ T cells in the liver of P-Tg compared with the control. **(C)** The proportion of CD45.1^+^ (donor) or CD45.2^+^ (host) in HBsAg dextramer^+^ CD8^+^ T cells in the liver of P-Tg. Data are shown as mean ± SEM. A Fisher’s exact test was used **(B)**. **P < 0.01. WT, WT control; Tg, HBs-Tg control; P-WT, WT parabiont; P-Tg, HBs-Tg parabiont.

By using HBsAg dextramer staining, HBsAg-specific CD8^+^ T cells were detected in the liver of HBs-Tg parabionts after 4w of parabiosis (**Figure 3A**). According to the data of the control WT group with nonspecific binding of dextramer reagents, a 95% confidence interval (CI) (gray dotted line, 0.16% ± 0.04%, n=6) was obtained, indicating that the frequency of HBsAg dextramer^+^CD8^+^ T cells (> 0.20%) was considered statistically positive (**Figure 3B**). HBsAg-specific CD8^+^ T cells were generated in all HBs-Tg parabionts but not in control HBs-Tg mice (**Figure 3B**). Notably, these HBsAg-specific CD8^+^ T cells were only detected in the liver of HBs-Tg parabionts but not in the WT parabionts (**Figure 3B**) and derived from WT mice (**Figure 3C**), indicating that donor HBsAg-specific CD8^+^ T cells can be induced while host HBsAg-specific CD8^+^ T cell clones will not be generated in the HBs-Tg parabionts.

### Donor HBsAg-specific CD8^+^ T cell response is primed in the spleen of HBs-Tg mice

**Figure 4:**
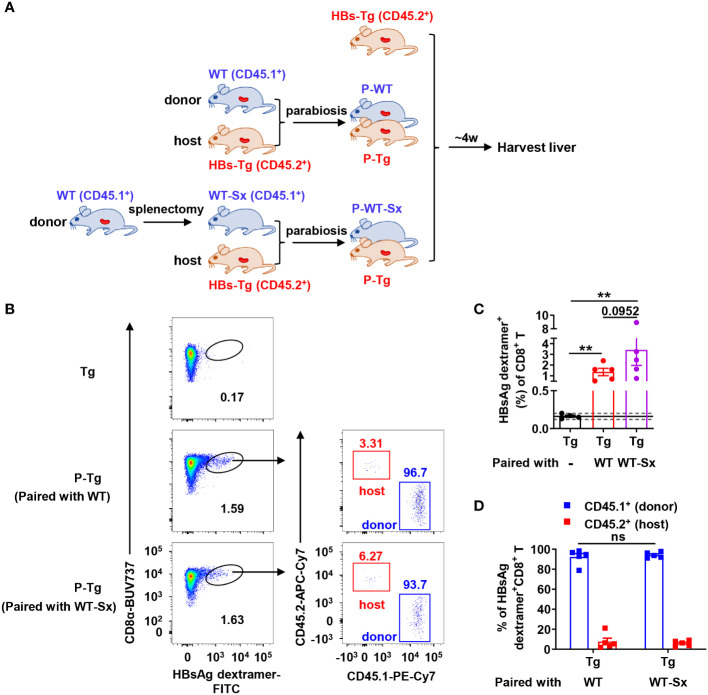
Donor HBsAg-specific CD8^+^ T cells could be generated in the HBs-Tg parabionts paired with splenectomized-WT mice. WT mice (CD45.1^+^) were performed splenectomy and were then surgically conjoined with HBs-Tg (CD45.2^+^) mice to generate WT-Sx-HBs-Tg parabiotic mice. Four weeks after parabiosis surgery, parabiotic mice were separated and liver samples were harvested from each group. Intrahepatic MNCs were isolated for flow cytometry analysis. **(A)** Protocol for the model of WT-Sx-HBs-Tg parabiotic mice and WT-HBs-Tg parabiotic mice (schematic). **(B)** Representative plots showing the frequency of HBsAg dextramer^+^CD8^+^ T cells in the liver of parabiotic and control HBs-Tg mice. **(C)** The frequency of HBsAg dextramer^+^CD8^+^ T cells in the liver of P-Tg (paired with WT-Sx), compared with P-Tg (paired with WT) and Tg (HBs-Tg control without parabiosis). The data ranging in 95% confidence interval (CI) (0.16% ± 0.04%, as described in **Figure 3B**) were considered negative. A Fisher’s exact test was used to compare the positive rate of MHC dextramer staining in P-Tg (paired with WT) and P-Tg (paired with WT-Sx) groups with Tg group respectively. The Mann-Whitney test was used to compare the frequency of HBsAg dextramer^+^ CD8^+^ T cells between P-Tg (paired with WT) and P-Tg (paired with WT-Sx) groups. **(D)** The proportion of CD45.1^+^ (donor) or CD45.2^+^ (host) in HBsAg dextramer^+^ CD8^+^ T cells from the liver of P-Tg (paired with WT-Sx), compared with P-Tg (paired with WT). Two-way ANOVA with Sidak’s multiple comparison test was used. Data are shown as mean ± SEM. The number between the bar show the p-value. **P < 0.01; ns, not significant. Tg, HBs-Tg control; P-WT, WT parabiont; P-Tg, HBs-Tg parabiont; Sx, splenectomy.

In the WT-HBs-Tg parabiotic model, there are two spleens: one is from WT mice and the other is from HBs-Tg mice. WT mice were performed splenectomy and then were conjoined with HBs-Tg mice by parabiosis surgery to generate WT-Sx-HBs-Tg parabiotic mice (**Figure 4A**). Compared with the HBs-Tg parabiont paired with WT, there was no significant difference in the frequency of HBsAg-specific CD8^+^ T cells in the liver of the HBs-Tg parabionts paired with WT-Sx mice, with a 100% positive rate (5/5) according to the 95% CI (0.16% ± 0.04%) of the negative control (**Figures 4B, C**). Furthermore, almost all HBsAg-specific CD8^+^ T cells were CD45.1 positive and derived from the donor WT-Sx mice (**Figures 4B, D**). These results demonstrated that the spleen of WT mouse was not essential to the generation of HBsAg-specific CD8^+^ T cells in this parabiotic model.

**Figure 5:**
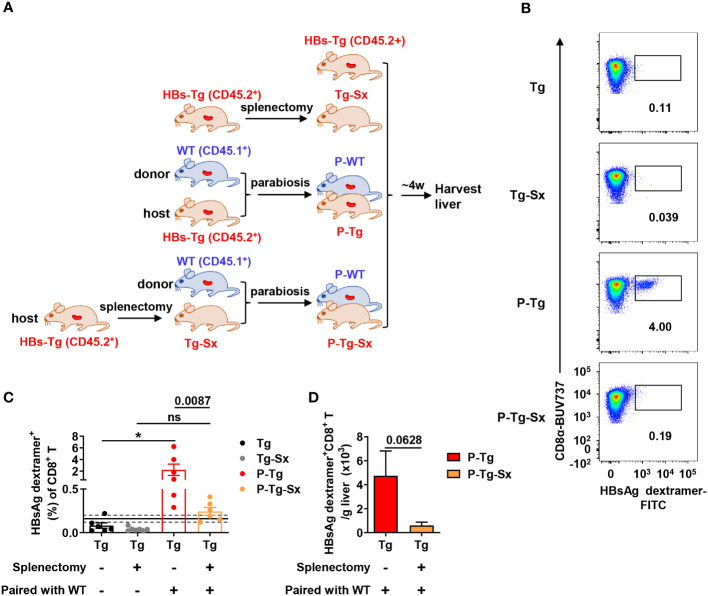
Donor HBsAg-specific CD8^+^ T cells could not be effectively generated in the splenectomized-HBs-Tg parabionts paired with WT mice. HBs-Tg mice (CD45.2^+^) were performed splenectomy, then were surgically conjoined with WT mice (CD45.1^+^) to generate WT-HBs-Tg-Sx parabiotic mice. Four weeks after surgery, parabiotic mice were separated and liver samples were harvested from each group. Intrahepatic MNCs were isolated for flow cytometry analysis. **(A)** Protocol for the model of WT-HBs-Tg-Sx parabiotic mice and WT-HBs-Tg parabiotic mice (schematic). **(B)** Representative plots showing the frequency of HBsAg dextramer^+^ CD8^+^ T cells in the liver of each group. **(C)** The frequency of HBsAg dextramer^+^ CD8^+^ T cells in the liver of P-Tg-Sx (splenectomized-HBs-Tg paired with WT), compared with P-Tg (HBs-Tg paired with WT), Tg (HBs-Tg control) and Tg-Sx (splenectomized-HBs-Tg control). The data ranging in 95% confidence interval (CI) (0.16% ± 0.04%, as described in **Figure 3B**) were considered negative. A Fisher’s exact test was used to compare the positive rate of MHC dextramer staining in P-Tg and P-Tg-Sx groups with the corresponding control group respectively. The Mann-Whitney test was used to compare the frequency of HBsAg dextramer^+^ CD8^+^ T cells between P-Tg and P-Tg-Sx groups. **(D)** The number of HBsAg dextramer^+^ CD8^+^ T cells in the liver of P-Tg and P-Tg-Sx. The Mann-Whitney test was used. Data are shown as mean ± SEM. The numbers between the bars show the p-values. *P < 0.05; ns, not significant. Tg, HBs-Tg control; P-WT, WT parabiont; P-Tg, HBs-Tg parabiont; Sx, splenectomy.

Similarly, HBs-Tg mice were performed splenectomy, and then were conjoined with WT mice by parabiosis surgery to generate WT-HBs-Tg-Sx parabiotic mice (**Figure 5A**). Notably, HBsAg-specific CD8^+^ T cells were hardly detected in the liver of splenectomized-HBs-Tg parabionts paired with WT mice (**Figure 5B**). The frequency of HBsAg-specific CD8^+^ T cells in the liver of splenectomized-HBs-Tg parabionts was significantly lower than that of HBs-Tg parabionts, and HBsAg-specific CD8^+^ T cells in the liver of splenectomized-HBs-Tg parabionts were only with the positive rate of 50% (3/6) (**Figure 5C**). The absolute number of HBsAg-specific CD8^+^ T cells was also decreased (**Figure 5D**). Overall, these results demonstrated that the spleen of HBs-Tg mouse was indispensable for the generation of HBsAg-specific CD8^+^ T cells, while the spleen of WT mouse was not in the WT-HBs-Tg parabiotic model.

### Donor CD4^+^ T cells are required for the priming of donor HBsAg-specific CD8^+^ T cells in the spleen of HBs-Tg mice

**Figure 6:**
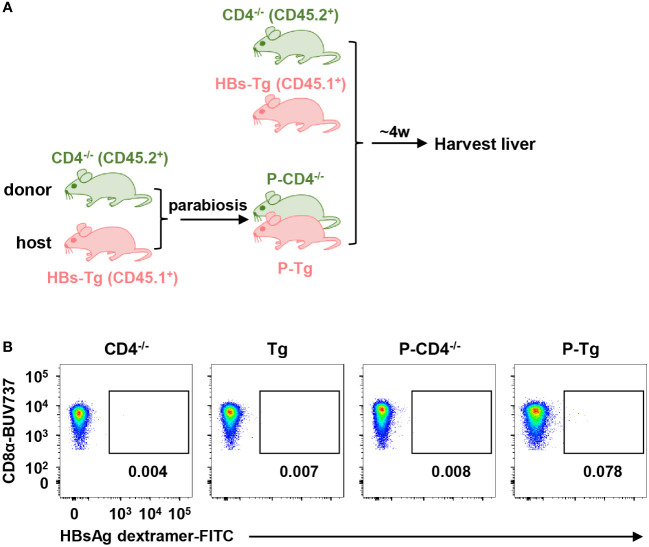
Donor HBsAg-specific CD8^+^ T cells could not be elicited in the HBs-Tg parabionts paired with CD4^-/-^ mice. HBs-Tg mice (CD45.1^+^) were surgically conjoined with CD4^-/-^ mice (CD45.2^+^) to generate CD4^-/-^-HBs-Tg parabiotic mice. Four weeks after surgery, parabiotic mice were separated and liver samples were harvested from each group. Intrahepatic MNCs were isolated for flow cytometry analysis. **(A)** Protocol for the model of CD4^-/-^-HBs-Tg parabiotic mice (schematic). **(B)** Representative plots showing the frequency of HBsAg dextramer^+^CD8^+^ T cells in the liver of each group. CD4^-/-^, CD4^-/-^ control; Tg, HBs-Tg control; P-CD4^-/-^, CD4^-/-^ parabiont; P-Tg, HBs-Tg parabiont.

To understand the role of donor CD4^+^ T (WT mice) and host CD4^+^ T (HBs-Tg mice) cells in the generation of HBsAg-specific CD8^+^ T cells in the WT-HBs-Tg parabiotic model, CD4 deficient mice were included. Firstly, CD4^-/-^ donor mice (CD45.2^+^) were conjoined with HBs-Tg mice (CD45.1^+^) by surgery to generate CD4^-/-^-HBs-Tg parabiotic mice, in which donor CD4^+^ T cells were deficient (**Figure 6A**). Indeed, no HBsAg-specific CD8^+^ T cells were detected in the HBs-Tg parabionts paired with CD4^-/-^ WT mice (**Figure 6B**), indicating that the priming of donor HBsAg-specific CD8^+^ T cells was dependent on donor CD4^+^ T cells in the HBs-Tg parabionts.

**Figure 7:**
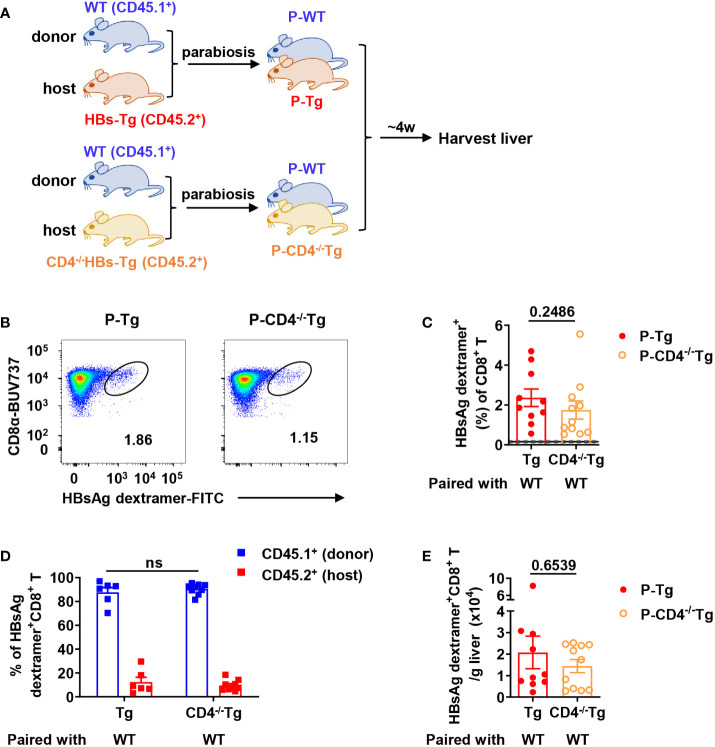
Donor HBsAg-specific CD8^+^ T cells were still generated in CD4^-/-^HBs-Tg parabionts paired with WT mice. CD4^-/-^HBs-Tg mice (CD45.2^+^) were surgically conjoined with WT mice (CD45.1^+^) to generate WT-CD4^-/-^HBs-Tg parabiotic mice. Four weeks after surgery, parabiotic mice were separated and liver samples were harvested from each group. Intrahepatic MNCs were isolated for flow cytometry analysis. **(A)** Protocol for the model of WT-CD4^-/-^HBs-Tg parabiotic mice (schematic). **(B)** Representative plots showing the frequency of HBsAg dextramer^+^CD8^+^ T cells in the liver of each group. **(C)** The frequency of HBsAg dextramer^+^ CD8^+^ T cells in the liver of P-CD4^-/-^Tg (paired with WT), compared with P-Tg (paired with WT). The data ranging in 95% confidence interval (CI) (0.16% ± 0.04%, as described in **Figure 3B**) were considered negative. **(D)** The proportion of CD45.1^+^ (donor) or CD45.2^+^ (host) in HBsAg dextramer^+^ CD8^+^ T cells from the liver of P-CD4^-/-^Tg, compared with P-Tg. **(E)** The number of HBsAg dextramer^+^ CD8^+^ T cells in the liver of P-Tg and P-CD4^-/-^Tg. The Mann-Whitney test was used **(C, E)**. Two-way ANOVA with Sidak’s multiple comparison test was used **(D)**. Data are shown as mean ± SEM. The numbers between the bars show the p-values. ns, not significant. P-Tg, HBs-Tg parabiont; P-CD4^-/-^Tg, CD4^-/-^HBs-Tg parabiont.

Secondly, WT mice (CD45.1^+^) were conjoined with CD4^-/-^HBs-Tg (CD45.2^+^) by surgery to generate WT-CD4^-/-^HBs-Tg parabiotic mice, in which host CD4^+^ T cells were deficient (**Figure 7A**). In this context, donor HBsAg-specific CD8^+^ T cells were still generated in the liver of CD4^-/-^HBs-Tg parabionts paired with WT mice (**Figures 7B–D**) and no significant difference was observed in the absolute number of HBsAg-specific CD8^+^ T cells between the HBs-Tg parabionts and CD4^-/-^HBs-Tg parabionts (**Figure 7E**). In the liver of CD4^-/-^HBs-Tg parabionts, the activation and effector functions of donor CD8^+^ T cells were not affected (**Figure S5)**. These results further demonstrated the sufficient help from donor CD4^+^ T cells played an important role in priming donor HBsAg-specific CD8^+^ T cells in the spleen of HBs-Tg parabionts.

### Existence of more tolerant host CD4^+^ Tregs in the spleen of HBs-Tg parabionts

It is necessary to determine why host CD4^+^ T (HBs-Tg mice) cells cannot help donor CD8^+^ T cell priming. Donor CD4^+^ T cells and host CD4^+^ T cells were compared in the spleen of HBs-Tg parabionts. The percentage of host CD4^+^ T cells was higher than that of donor CD4^+^ T cells (**Figures S6A, B**). And the ratio of host CD4^+^ T cells to CD8^+^ T cells was also higher than that of donor (**Figure S6C**). Compared with donor CD4^+^ T cells, the higher expression level of TIGIT was observed on host CD4^+^ T cells, but not CTLA-4, PD-1 and Tim-3 (**Figures S6D, E**); however, the numbers of host CTLA-4^+^, PD-1^+^ and TIGIT^+^ CD4^+^ T cells were more than those of the donor (**Figure S6F**). These results indicated that host CD4^+^ T cells may be more suppressive than donor CD4^+^ T cells.

**Figure 8:**
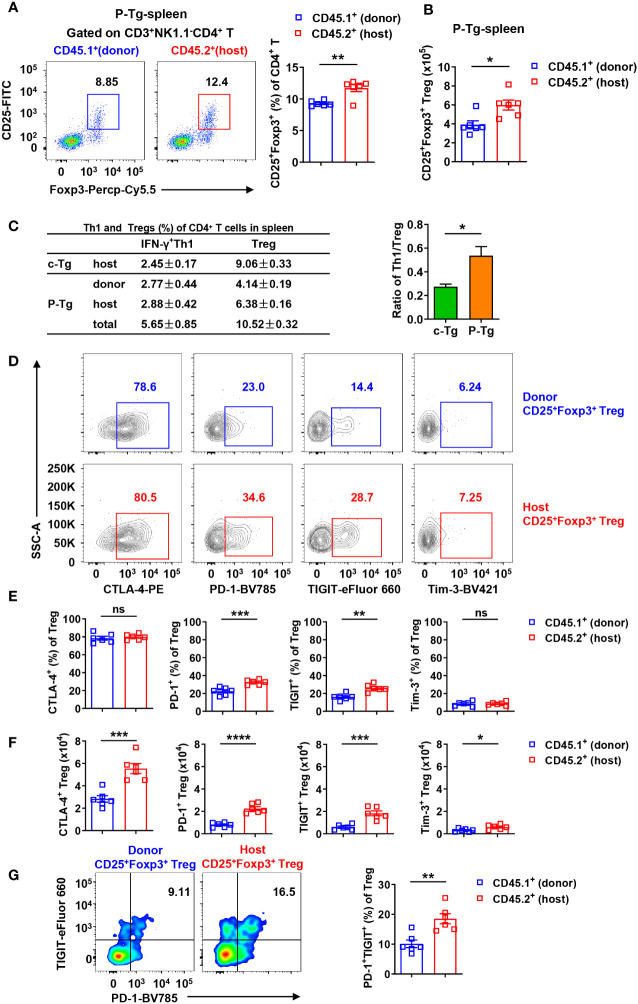
Host CD25^+^Foxp3^+^ Tregs were tolerant with higher expression levels of PD-1 and TIGIT in the spleen of HBs-Tg parabionts. WT-HBs-Tg parabiotic mice were generated by parabiosis surgery as shown in **Figure 1A**. Splenic MNCs were isolated from Tg and P-Tg mice for flow cytometry analysis after 4 weeks of parabiosis. **(A)** Donor CD45.1^+^CD3^+^NK1.1^-^CD4^+^ T cells and host CD45.2^+^CD3^+^NK1.1^-^CD4^+^ T cells were gated to analyze the proportion of CD25^+^Foxp3^+^ Tregs in the spleen of P-Tg mice. **(B)** The number of donor and host CD25^+^Foxp3^+^ Tregs in the spleen of P-Tg mice. **(C)** The frequencies of donor or host IFN-γ^+^CD4^+^ T cells (Th1) and CD25^+^Foxp3^+^ Tregs among total CD4^+^ T cells in the spleen of Tg and P-Tg mice. Data are shown as mean ± SEM in each group. The ratio of total Th1/Treg in the spleen was compared between Tg mice and P-Tg mice. **(D)** Representative plots showing the expression of CTLA-4, PD-1, TIGIT and Tim-3 in donor CD4^+^CD25^+^Foxp3^+^ Tregs or host CD4^+^CD25^+^Foxp3^+^ Tregs in the spleen of P-Tg mice. **(E)** Statistical analysis of the percentages of CTLA-4, PD-1, TIGIT and Tim-3 on Tregs from **(D)**. **(F)** Number of CTLA-4, PD-1, TIGIT or Tim-3 positive Tregs from donor and host in the spleen of P-Tg mice. **(G)** The coexpression of PD-1 and TIGIT in donor CD4^+^CD25^+^Foxp3^+^ Tregs and host CD4^+^CD25^+^ Foxp3^+^ Tregs in the spleen of P-Tg mice. Data are shown as mean ± SEM. An unpaired two-tailed t-test was used. *P < 0.05; **P < 0.01; ***P < 0.001; ****P < 0.0001; ns, not significant. Tg, HBs-Tg control; P-Tg, HBs-Tg parabiont.

Further, a higher frequency and more absolute number of host CD25^+^Foxp3^+^ Tregs were observed in the spleen of HBs-Tg parabionts (**Figures 8A, B**). Then we analyzed the frequencies of IFN-γ^+^ Th1 cells and Tregs of total CD4^+^ T cells in the spleen between donor and host and found that the ratio of total Th1/Treg in the spleen was markedly enhanced from 0.27 ± 0.02 in control HBs-Tg to 0.54 ± 0.08 in HBs-Tg parabionts, indicating a possibility that increased donor Th1 cells overcame the inhibition of Tregs in the host spleen of HBs-Tg parabionts (**Figure 8C**). Compared with donor CD25^+^Foxp3^+^ Tregs, much higher expression levels of PD-1 and TIGIT were detected on host CD25^+^Foxp3^+^ Tregs in the spleen of HBs-Tg parabionts, whereas no significant differences in CTLA-4 and Tim-3 (**Figures 8D, E**). Notably, the numbers of host CTLA-4^+^, PD-1^+^, TIGIT^+^ and Tim-3^+^ populations of CD25^+^Foxp3^+^ Tregs were much more than those of donor CD25^+^Foxp3^+^ Tregs (**Figure 8F**), especially the host PD-1^+^TIGIT^+^ Tregs (**Figure 8G**), indicating that more host Tregs with stronger suppressive functions were involved in the failure of HBsAg-specific CD8^+^ T cell priming.

**Figure 9:**
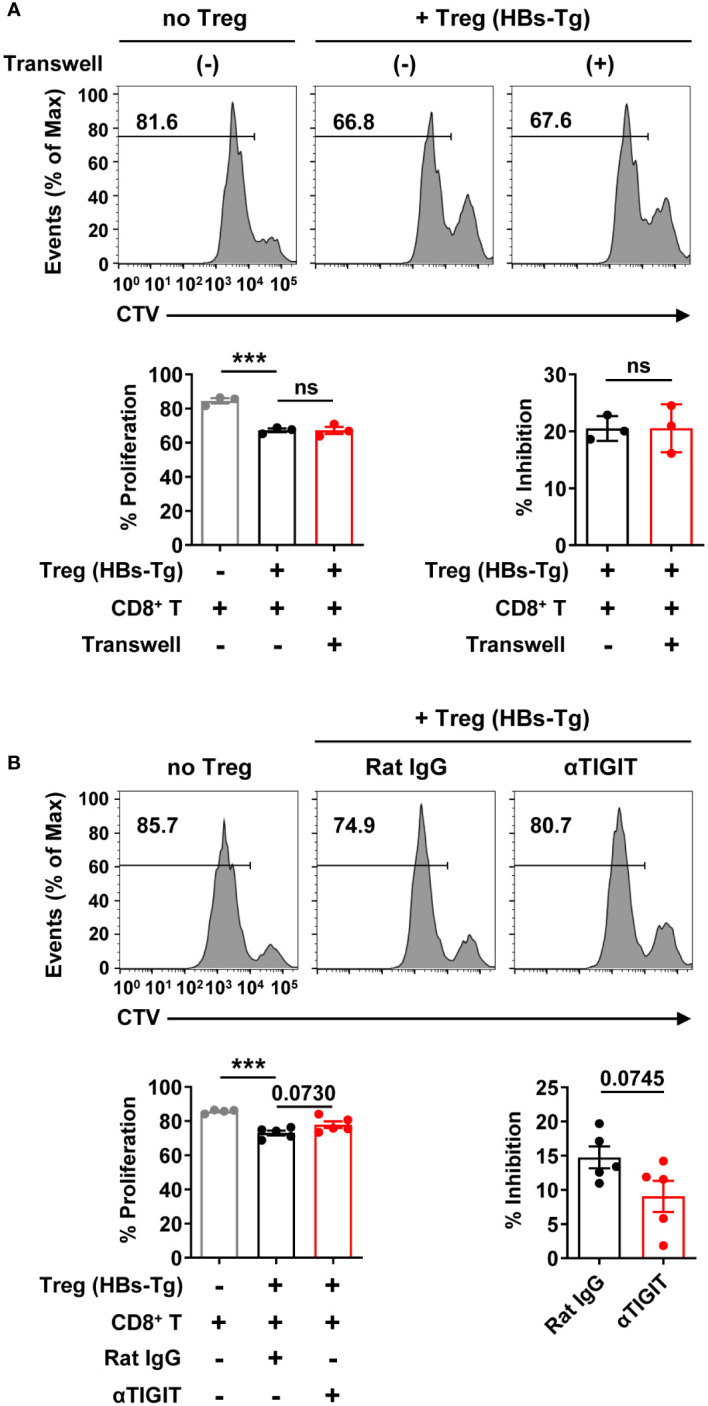
Tregs inhibited CD8^+^ T cell proliferation involving TIGIT molecule. **(A)** The CTV-labeled CD8^+^ T cells from the spleens of WT B6 mice (CD45.1^+^) were plated on 24-well plate (1×10^6^/well) and then stimulated with anti-CD3 mAb (2 μg/mL), anti-CD28 mAb (1 μg/mL), and IL-2 (100 IU/mL). CD4^+^CD25^+^ Tregs cells from the spleens of HBs-Tg mice were co-cultured at a ratio of 1:2 to CD8^+^ T cells for 3 days in the absence or presence of transwell membranes (0.4 μm pore size). Statistical results are shown at the bottom. **(B)** The CTV-labeled CD8^+^ T cells were plated on 96-well plate (1×10^5^/well) and then stimulated with anti-CD3 mAb (2 μg/mL), anti-CD28 mAb (1 μg/mL), and IL-2 (100 IU/mL). CD4^+^CD25^+^ Tregs cells were co-cultured at a ratio of 1:1 to CD8^+^ T cells for 3 days in the presence of Rat IgG or αTIGIT (10 μg/mL). Statistical results are shown at the bottom. Data are shown as mean ± SEM. One-way ANOVA with Sidak’s multiple comparison test or an unpaired two-tailed t-test were used. The numbers between the bars show the p-values. ***P < 0.001; ns, not significant.

To further investigate whether and how Tregs from HBs-Tg mice suppress CD8^+^ T cell response, Tregs and CD8^+^ T cells co-cultured experiments were performed *in vitro*. Tregs from HBs-Tg mice displayed significant suppressive capacity for the proliferation of CD8^+^ T cells (**Figure 9A**). When the Tregs and CD8^+^ T cells were separated by the transwell membrane, the inhibitory role of Tregs on CD8^+^ T cells was still observed, indicating that direct cell-cell contact was not necessary (**Figure 9A**). When TIGIT was blocked by anti-TIGIT mAb, Tregs showed decreased inhibition of the proliferation of CD8^+^ T cells (**Figure 9B**), indicating the involvement of TIGIT in the suppressive function of Tregs.

## Discussion

**Figure 10:**
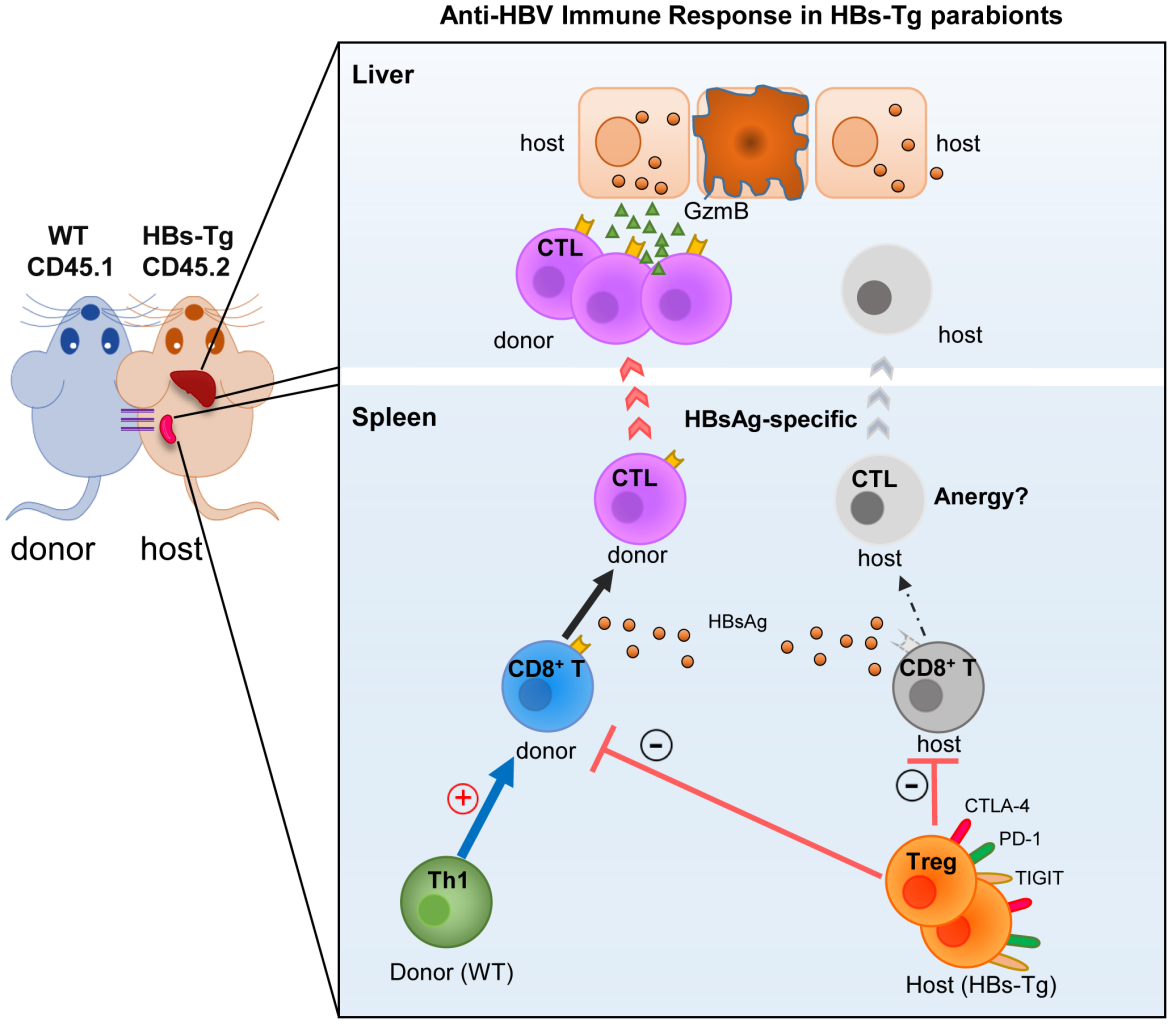
Mechanisms of HBV immune tolerance in HBs-Tg parabionts. By using the WT-HBs-Tg parabiotic mouse model, the major mechanisms underlying the immune tolerance of HBV persistence were elucidated, including the incompetent anti-HBV CD8^+^ T cells and insufficient help from CD4^+^ T cells in HBs-Tg mice.

In this study, by using a WT (donor)-HBs-Tg (host) parabiotic mice model, the mechanisms of HBV immune tolerance were elucidated more clearly. It was observed that donor HBsAg-specific CD8^+^ T cells were elicited effectively in the liver of HBs-Tg parabionts, which were primed in the spleen of HBs-Tg, but not WT mice, with efficient help from donor CD4^+^ T cells. Notably, host CD4^+^ T cells harboring more Tregs with higher expression of TIGIT and PD-1 inhibitory molecules played a suppressive role in this process by inhibiting the activation of donor CD8^+^ T cells and maintaining the tolerance of host CD8^+^ T cells. These results revealed the major mechanisms underlying immune tolerance in HBs-Tg mice, including incompetent anti-HBV CD8^+^ T cells and insufficient help from CD4^+^ T cells, which helps explain HBV persistence (**Figure 10**).

CD8^+^ T and CD4^+^ T cells were responsible for HBV immune tolerance; however, the precise cellular mechanisms were not elucidated in detail previously due to the limitation of the HBV model. Because of the central and peripheral tolerance to HBV in HBV-Tg mice, the direct adoptively transferred naïve CD8^+^ TCR transgenic T cells could not be functionally activated, and also dendritic cell (DC) immunization did not induce an efficient anti-HBV CTL response (38), thus indicating that the anti-HBV CD8^+^ T cell immune response could not be explored in HBV-Tg mice (39, 40). Another commonly used HBV mouse model is HBV-carrier mice based on hydrodynamic HBV plasmid transfection, which closely mimics human asymptomatic HBV carriers (41). Short persistence with only a 10% HBV positive rate of hepatocytes and HBV immune tolerance make this model unsuitable to see how the immune system naturally responds to HBV. Our previously generated HBs-HepR mouse model transferred with HBsAg^+^ hepatocytes from HBs-Tg mice in immunocompetent Fah^-/-^ mice also has the limitation that the liver inflammation mediated by hepatocyte damage of recipient Fah^-/-^ mice would affect the exploration of the natural immune response to HBV (33, 34). Immunocompetent human liver chimeric mice have a great advantage in studying the host adaptive immune responses to HBV; however, the need for primary human hepatocytes and immune cells or fetal/cord blood cells and the complex approach greatly limit their application (42).

In this study, WT-HBs-Tg parabiotic mice model can avoid these limitations. HBsAg could be secreted into blood by hepatocytes of HBs-Tg mouse and persistently existed in peripheral blood, mimicking the persistent blood-born HBsAg in patients with chronic HBV infection. Moreover, naïve immune system from WT mouse was responsive to HBV. These render the WT-HBs-Tg parabiotic mice model closer to the natural HBV infection in humans. Thus, WT-HBs-Tg parabiotic mice model is better to study how to naturally respond to HBV from immunocompetent WT mice and how to influence the anti-HBV immunity by tolerant HBs-Tg mice. Donor HBsAg-specific CTL response was triggered by the blood-born HBsAg in host HBs-Tg mice (**Figure 3**), but not the humoral immune response with a low positive rate (3/14) of anti-HBs (data not shown), which was consistent with the observation in HBV-infected patients that HBV-specific CTLs are important in the anti-HBV immune response and critical to HBV clearance (12, 43). By using this WT-HBs-Tg parabiotic model, we clarified the previously unvalidated immunological mechanisms of the HBV immune response which are not easy to observe in other HBV mouse models.

We are also interested in the mechanism by which CD8^+^ T cells can be primed in the spleen of HBs-Tg mice rather than in the spleen of WT mice. Splenic DCs from HBV-Tg mice displayed normal Ag processing and presenting activities *in vitro* (38), suggesting that the spleen of HBs-Tg mice could be a normal site of CD8^+^ T cell priming. HBV-specific CD4^+^ T cells are considered to be important facilitators for inducing and maintaining CD8^+^ T cells (21, 22). In this study, we took advantage of splenectomy to reveal that the spleen of HBs-Tg mice, but not the spleen of WT mice, was the essential site for CD8^+^ T cell priming in the WT-HBs-Tg parabiotic model (**Figures 4**; **5**). Donor CD4^+^ T cell help was essential to HBV-specific CD8^+^ T cell priming in the spleen (**Figure 6**). A novel protein-prime/MVA-boost vaccination could break HBV-specific immune tolerance in HBV-Tg mice. In this process, protein priming of the HBV-specific CD4^+^ T cell response was initiated, which was suspected to provide help for CD8^+^ T cell responses (44). A few clinical trials of therapeutic vaccination to HBV provided relevant perceptions into the importance of CD4^+^ T cell help. The failure of clinical trials of therapeutic vaccines such as GS-4774 may be explained by the absence of therapeutic vaccine-induced CD4^+^ T cells, as exclusively CD8^+^ T cell responses were triggered (45). Thus, CD4^+^ T cell-mediated help has a great influence on the outcome of CD8^+^ T cell responsiveness.

HBsAg-specific CD8^+^ T cells can be elicited in the periphery of adult HBs-Tg mice by, e.g., DC immunization or TIGIT blockade, possibly reflecting that HBs-Tg mice harbor peripheral tolerance (14, 38). Here, it was demonstrated that host tolerant CD4^+^ T cells maintained the peripheral immune tolerance of HBsAg-specific CD8^+^ T cells (**Figure S6**). There was a group of Tregs with high expression of CTLA-4, PD-1 and TIGIT inhibitory receptors (**Figures 8D–G**), which accounted for the suppression of the CD8^+^ T response (**Figure 9**). As reported (46), TIGIT was crucial to maintain Treg suppressive function (**Figure 9B**). Expression of PD-1 on Tregs was also necessary to suppress CD8^+^ T cells during chronic LCMV infection (47). Recently, PD-L1 dependent regulation on HBsAg-specific CD8^+^ T cells was demonstrated for liver-resident NK cells in chronic HBV infection (48). However, PD-1 was also demonstrated to inhibit Tregs, since PD-L1 blockade could ameliorate PD-1^hi^ Treg depletion and enhance their functions during infection with Toxoplasma gondii (49); and selective deficiency of PD-1 in Tregs augmented their immunosuppressive functions in experimental autoimmune encephalomyelitis (50). Thus, the precise mechanisms of Treg regulation on CD8^+^ T cells deserve further investigation. Patients with chronic HBV infection have higher levels of CD4^+^ CD25^+^ Tregs and more defective HBV-specific CD8^+^ T cells than patients spontaneously recover (51, 52). Tregs could inhibit the HBV-specific CD8^+^ T cell responses, and positively correlated with serum HBV load (29, 53). A clinical study of patients with chronic HBV infection also showed that reduction of Tregs by the combination of GS-4774 and tenofovir disoproxil fumarate (TDF) therapy efficiently induced CD8^+^ T cell responses (45).

In this study, our results strongly support that efficient donor CD4^+^ T cell help can overcome the suppression of host Tregs and induce HBsAg-specific CD8^+^ T cells (**Figure 8C**). Thus, both Th1 cells and Tregs exhibit indispensable regulation on the CTL response and the balance of them determines the outcome of the anti-HBV CTL response. HBV-specific Tregs might contribute to the suppression of the HBV-specific Th1 cell response in patients with chronic HBV infection (26, 27). Tregs can suppress the proinflammatory Th1 response through the secretion of Fgl2 induced by engagement of TIGIT (54), and also suppress the proinflammatory cytokine IL-12 but increase the cytokine IL-10 in DCs through the TIGIT-CD155 interaction between Tregs and DCs, thereby inhibiting T cell activation (55). The interaction between Tregs and Th1 cells deserves further investigation to clarify the peripheral immune tolerance of HBV.

In conclusion, by using the WT-HBs-Tg parabiotic mouse model, the major mechanisms underlying the immune tolerance of HBV persistence were elucidated, including the incompetent anti-HBV CD8^+^ T cells and insufficient help from CD4^+^ T cells in HBs-Tg mice. These results provide a theoretical basis for developing effective immunotherapeutic strategies against chronic HBV infections.

## Data availability statement

The original contributions presented in the study are included in the article/**Supplementary Materials**. Further inquiries can be directed to the corresponding authors.

## Ethics statement

The animal study was reviewed and approved by The Ethics Committee for Animal Care and Use of the University of Science and Technology of China.

## Author contributions

YC, RS, and ZT initiated and designed the research. WZ performed all the experiments and analyzed and interpreted the results. YC, WZ, and ZT wrote the manuscript. HW contributed to the discussion of the results. All authors contributed to the article and approved the submitted version.

## Funding

This work was supported by National Key R&D Program of China (2021YFC2300601/4, 2018YFA0507403), Chinese Academy of Science (XDB29030201) and Natural Science Foundation of China (81788101, 82071764, 91842307).

## Conflict of interest

The authors declare that the research was conducted in the absence of any commercial or financial relationships that could be construed as a potential conflict of interest.

## Publisher’s note

All claims expressed in this article are solely those of the authors and do not necessarily represent those of their affiliated organizations, or those of the publisher, the editors and the reviewers. Any product that may be evaluated in this article, or claim that may be made by its manufacturer, is not guaranteed or endorsed by the publisher.

## Abbreviation

HCC, hepatocellular carcinoma; HBV, hepatitis B virus; CHB, chronic hepatitis B; HBsAg, hepatitis B surface antigen; HBcAg, hepatitis B core antigen; MNCs, mononuclear cells; CTL, cytotoxic T lymphocyte; Treg, regulatory T cell; Th1, type 1 helper T cell; KC, Kupffer cell; APC, antigen-presenting cell; MDSC, myeloid-derived suppressor cell; NK, natural killer cell; IFN-γ, interferon-γ; TNF-α, tumor necrosis factor-α; WT: wild type; HBs-Tg, HBs-transgenic mice; HBV-Tg, HBV transgenic mice; DC, dendritic cell.
