# Supplementary material for: HBV immune tolerance of HBs-transgenic mice observed through parabiosis with WT mice
Source: Front Immunol. 2022 Sep 20;13:993246. doi: 10.3389/fimmu.2022.993246 (PMC9530942; doi:10.3389/fimmu.2022.993246)
Supplement: Supplementary file 1 [file DataSheet_1.docx]

**Supplementary Figures**


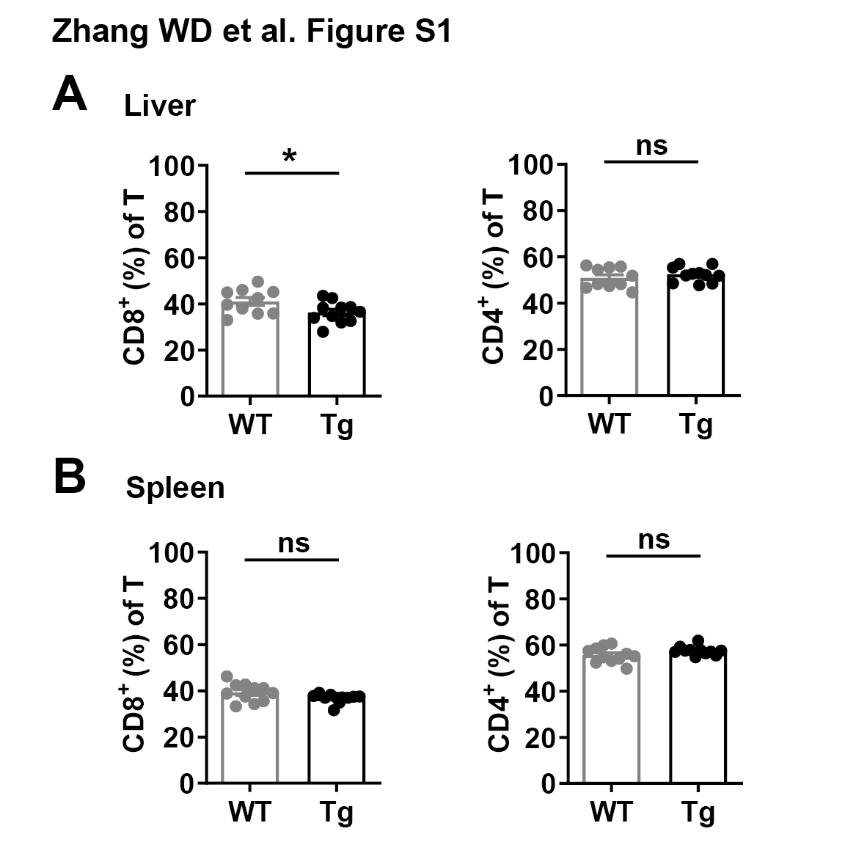


**Supplementary Figure 1. The frequencies of CD8^+^ T cells and CD4^+^ T cells were compared between HBs-Tg mice and WT mice.** Mononuclear cells (MNCs) were isolated from the liver and spleen of WT mice and HBs-Tg mice and then analyzed by flow cytometry. (A) The percentages of CD8^+^ T and CD4^+^ T of total T cells (CD45^+^CD3^+^NK1.1^-^) in the liver. (B) The percentages of CD8^+^ T and CD4^+^ T of total T cells in the spleen. Data are shown as mean ± SEM. An unpaired two-tailed t-test was used. **P* < 0.05; ns, not significant.

**
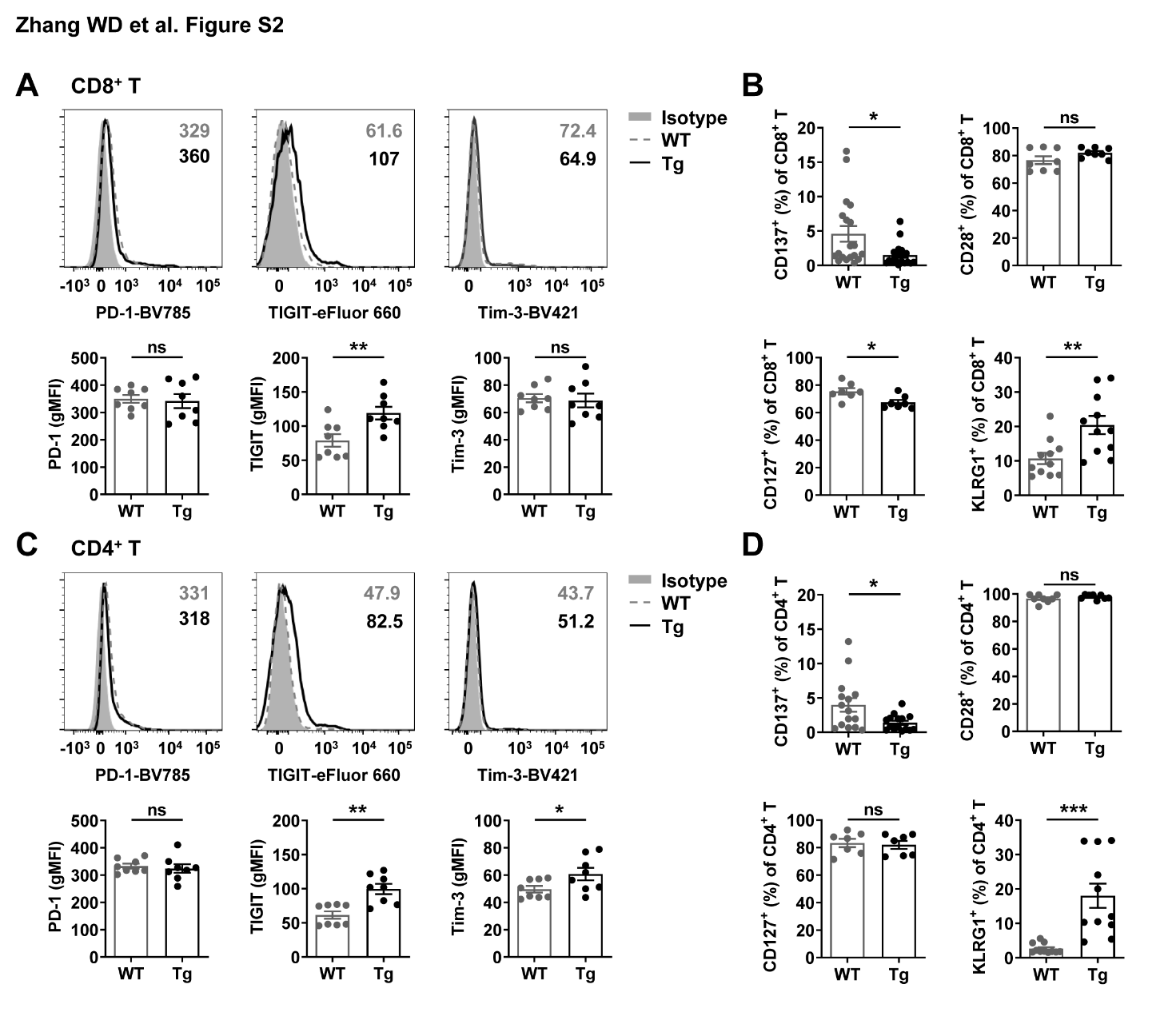
**

**Supplementary Figure 2. Hepatic CD8^+^ T and CD4^+^ T cells were dysfunctional in the HBs-Tg mice.** Mononuclear cells (MNCs) were isolated from the livers of WT mice and HBs-Tg mice and then analyzed by flow cytometry. (A, C) Representative histograms (top) and gMFI (bottom) of PD-1, TIGIT and Tim-3 expression on CD8^+^ T cells (A) and CD4^+^ T cells (C) from WT mice and HBs-Tg mice. (B, D) The expression of CD137, CD28, CD127, and KLRG1 on intrahepatic CD8^+^ T cells (B) and CD4^+^ T cells (D) of HBs-Tg mice compared with WT mice. Data are shown as mean ± SEM. An unpaired two-tailed t-test was used. **P* < 0.05; ***P* < 0.01; ****P* < 0.001; ns, not significant.


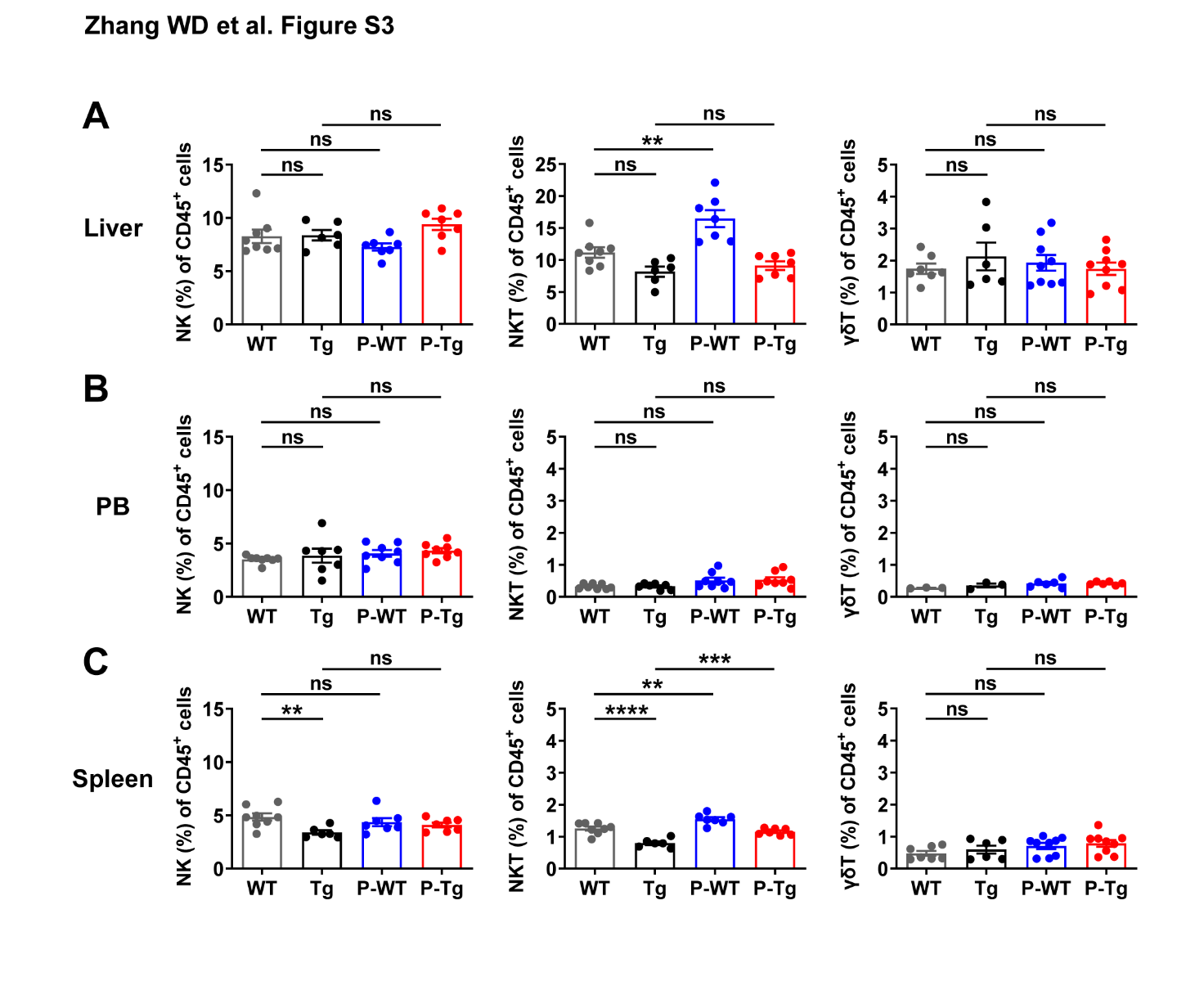


**Supplementary Figure 3. Frequencies of NK, NKT and γδT cells in the liver, peripheral blood, and spleen of HBs-Tg parabionts compared with the control.** WT-HBs-Tg parabiotic mice were generated by parabiosis surgery as shown in Figure 1A. Four weeks after parabiosis, liver, peripheral blood (PB), and spleen samples were harvested. Mononuclear cells (MNCs) were isolated for flow cytometry analysis. The frequencies of NK cells (CD45^+^CD3^-^NK1.1^+^), NKT cells (CD45^+^CD3^+^NK1.1^+^), and γδT cells (CD45^+^CD3^+^γδTCR^+^) were shown in the liver, peripheral blood, and spleen at 4 weeks after parabiosis. Data are shown as mean ± SEM. One-way ANOVA with Sidak’s multiple comparison test was used to compare the experimental groups. ***P* < 0.01; ****P* < 0.001; *****P* < 0.0001; ns, not significant. WT, WT control; Tg, HBs-Tg control; P-WT, WT parabiont; P-Tg, HBs-Tg parabiont.

**
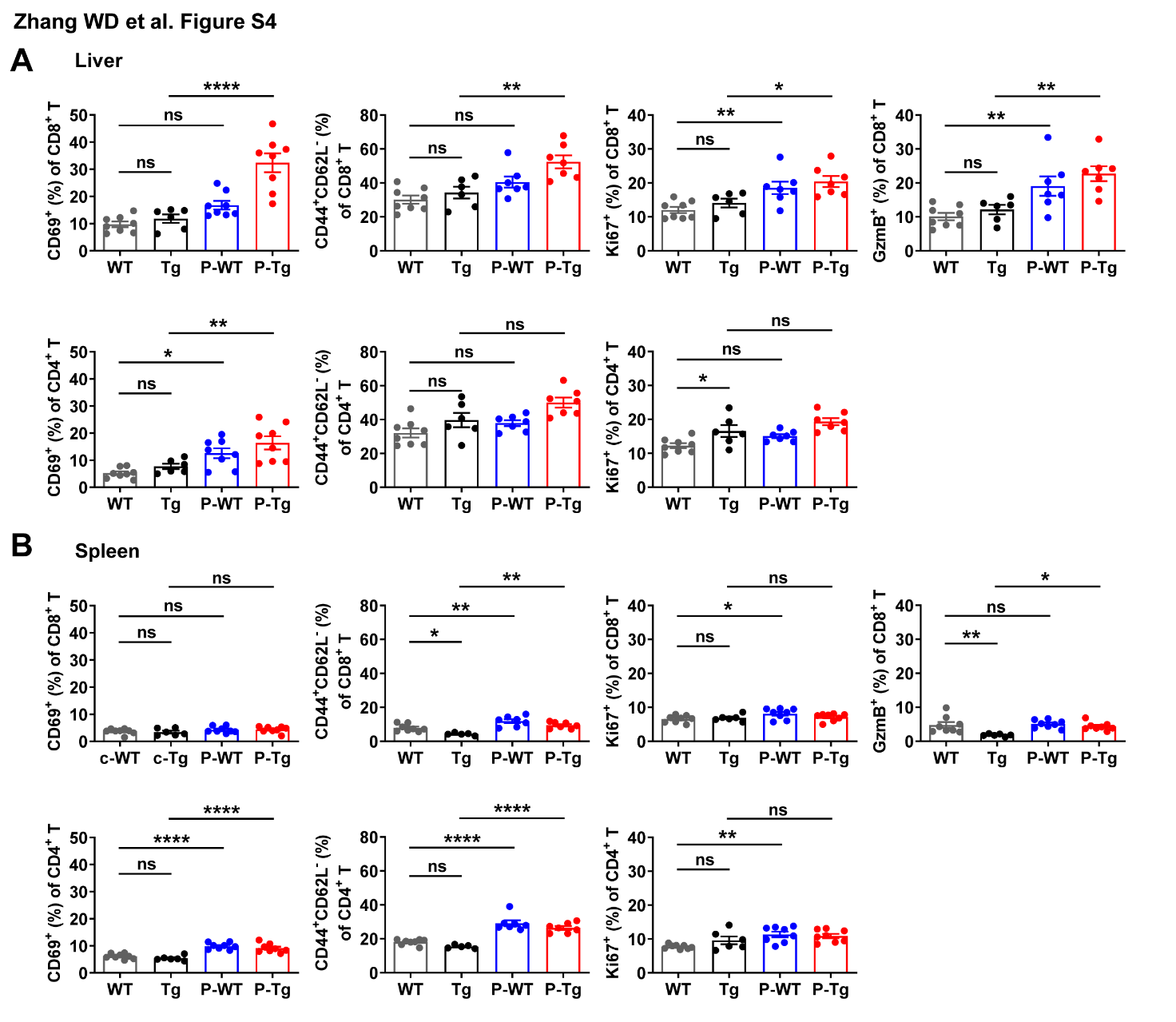
**

**Supplementary Figure 4. CD8^+^ T and CD4^+^ T cells were functionally activated in the liver and spleen of WT and HBs-Tg mice after parabiosis.** WT-HBs-Tg parabiotic mice were generated by parabiosis surgery as shown in Figure 1A. Four weeks after parabiosis, liver and spleen samples were harvested. Mononuclear cells (MNCs) were isolated for flow cytometry analysis. Frequencies of phenotypes (CD69, CD44^+^CD62L^-^, Ki67 and Granzyme B) on CD8^+^ T and CD4^+^ T cells in the liver (A) and spleen (B). Data are shown as mean ± SEM. One-way ANOVA with Sidak’s multiple comparison test was used to compare the experimental groups. **P* < 0.05; ***P* < 0.01; *****P* < 0.0001; ns, not significant. WT, WT control; Tg, HBs-Tg control; P-WT, WT parabiont; P-Tg, HBs-Tg parabiont.


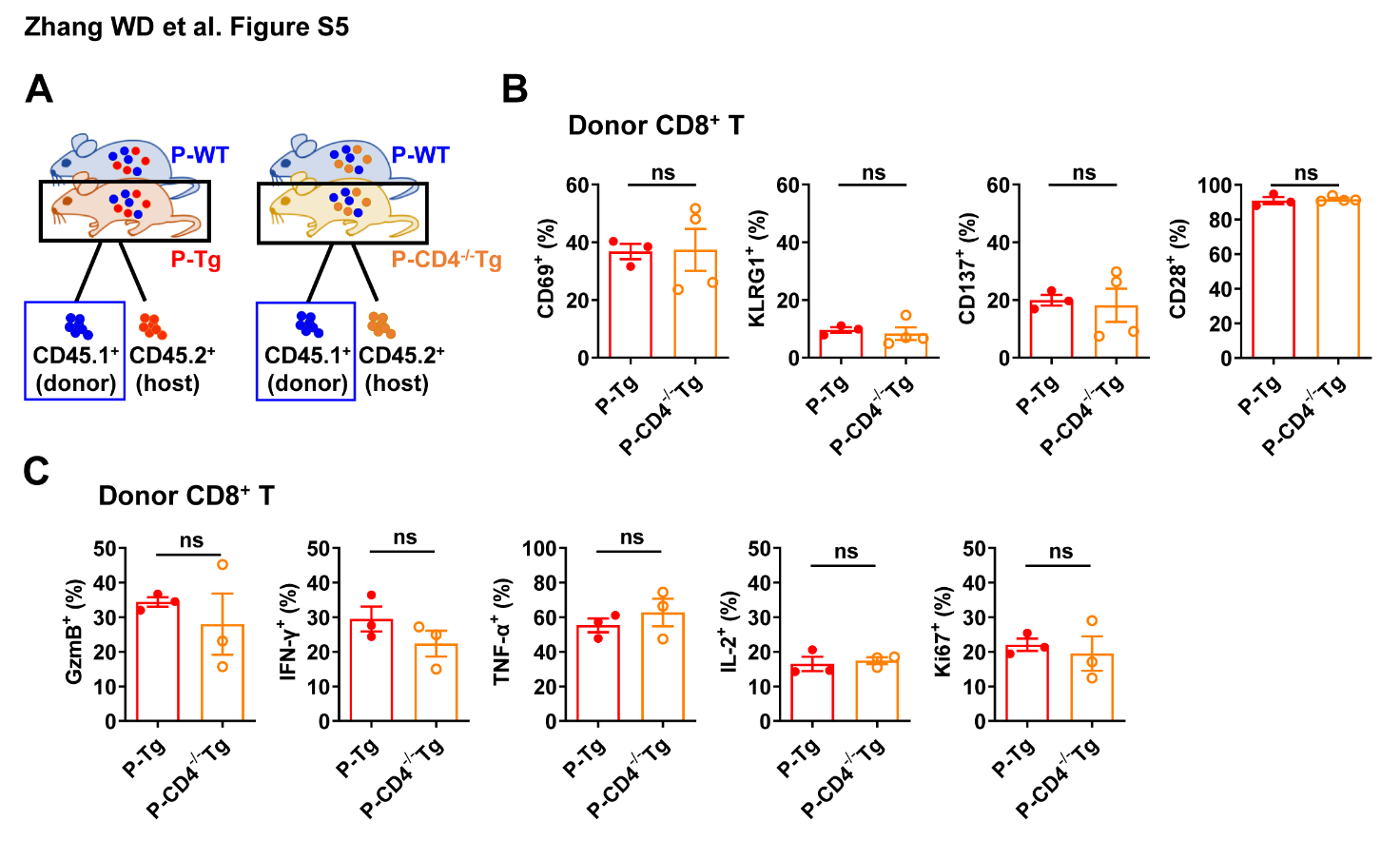


**Supplementary Figure 5. No significant differences in the activation and effector functions of donor CD8^+^ T cells in CD4^-/-^HBs-Tg parabionts compared with HBs-Tg parabionts.** (A) Intrahepatic MNCs were isolated from P-Tg and P- CD4^-/-^Tg mice for flow cytometry analysis after 4 weeks of parabiosis. Donor CD45.1^+^CD3^+^NK1.1^-^CD8^+^ T cells were gated for analysis (schematic). (B) The expression of CD69, KLRG1, CD137 and CD28 on donor CD45.1^+^CD3^+^NK1.1^-^CD8^+^ T cells from the liver of P-CD4^-/-^Tg compared with P-Tg. (C) The expression of Granzyme B, IFN-γ, TNF-α, IL-2 and Ki67 in donor CD45.1^+^CD3^+^NK1.1^-^CD8^+^ T cells from the liver of P-CD4^-/-^Tg compared with P-Tg. Data are shown as mean ± SEM. An unpaired two-tailed t-test was used to compare the experimental groups. ns, not significant. P-WT, WT parabiont; P-Tg, HBs-Tg parabiont; P-CD4^-/-^Tg, CD4^-/-^HBs-Tg parabiont.


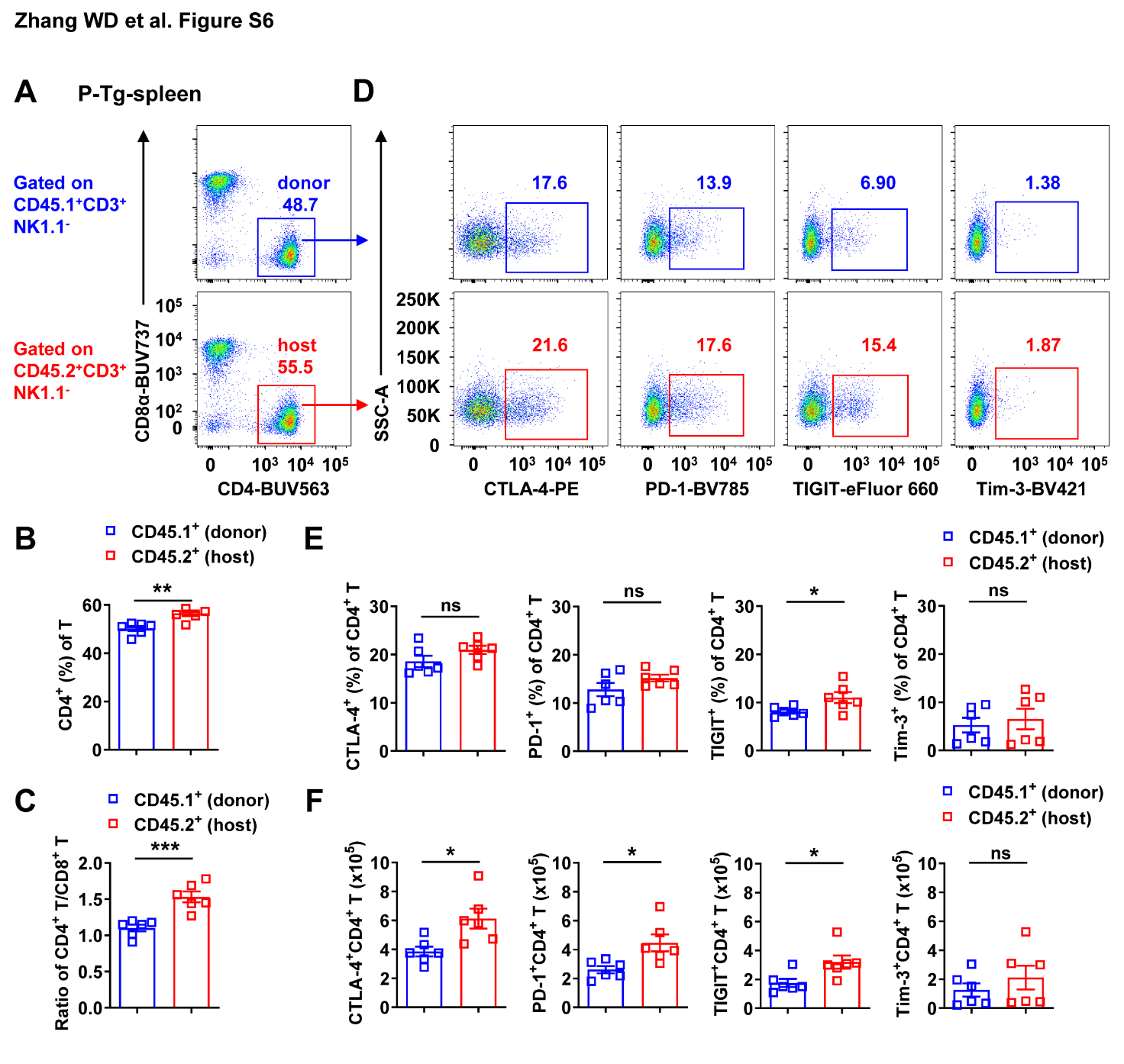


**Supplementary Figure 6. More host CD4^+^ T cells were with inhibitory phenotypes than donor CD4^+^ T cells in the spleen of HBs-Tg parabionts.** WT-HBs-Tg parabiotic mice were generated by parabiosis surgery as shown in Figure 1A. Splenic MNCs were isolated from P-Tg mice for flow cytometry analysis after 4 weeks of parabiosis. (A, B, C) Donor CD45.1^+^CD3^+^NK1.1^-^ and host CD45.2^+^CD3^+^NK1.1^-^ cells were gated to analyze the proportion of CD4^+^ T cells and the ratio of CD4^+^ T/CD8^+^ T cells, respectively. (D) Representative plots showing the expression of CTLA-4, PD-1, TIGIT and Tim-3 in donor CD4^+^ T or host CD4^+^ T cells. (E, F) Comparison of the percentage and number of the indicated CD4^+^ T cell subsets between donor and host. Data are shown as mean ± SEM. An unpaired two-tailed t-test was used. **P* < 0.05; ***P* < 0.01; ****P* < 0.001; ns, not significant. P-Tg, HBs-Tg parabiont.
